# Epi-mutations for spermatogenic defects by maternal exposure to di(2-ethylhexyl) phthalate

**DOI:** 10.7554/eLife.70322

**Published:** 2021-07-28

**Authors:** Yukiko Tando, Hitoshi Hiura, Asuka Takehara, Yumi Ito-Matsuoka, Takahiro Arima, Yasuhisa Matsui

**Affiliations:** 1 Cell Resource Center for Biomedical Research, Institute of Development, Aging and Cancer, Tohoku UniversitySendaiJapan; 2 Graduate School of Life Sciences, Tohoku UniversitySendaiJapan; 3 Department of Bioscience, Faculty of Life Sciences, Tokyo University of AgricultureTokyoJapan; 4 Department of Informative Genetics, Environment and Genome Research Center, Graduate School of Medicine, Tohoku UniversitySendaiJapan; 5 Graduate School of Medicine, Tohoku UniversitySendaiJapan; University of Southern CaliforniaUnited States; Weill Cornell MedicineUnited States

**Keywords:** germ cell, spermatogenesis, DNA methylation, Di (2-ethylhexyl) phthalate, Mouse

## Abstract

Exposure to environmental factors during fetal development may lead to epigenomic modifications in fetal germ cells, altering gene expression and promoting diseases in successive generations. In mouse, maternal exposure to di(2-ethylhexyl) phthalate (DEHP) is known to induce defects in spermatogenesis in successive generations, but the mechanism(s) of impaired spermatogenesis are unclear. Here, we showed that maternal DEHP exposure results in DNA hypermethylation of promoters of spermatogenesis-related genes in fetal testicular germ cells in F1 mice, and hypermethylation of *Hist1h2ba*, *Sycp1,* and *Taf7l*, which are crucial for spermatogenesis, persisted from fetal testicular cells to adult spermatogonia, resulting in the downregulation of expression of these genes. Forced methylation of these gene promoters silenced expression of these loci in a reporter assay. These results suggested that maternal DEHP exposure-induced hypermethylation of *Hist1h2ba*, *Sycp1,* and *Taf7l* results in downregulation of these genes in spermatogonia and subsequent defects in spermatogenesis, at least in the F1 generation.

## Introduction

A wide variety of environmental influences have been shown to greatly impact human health, with the effects persisting through multiple generations (Daxinger and Whitelaw, 2012; Taouk and Schulkin, 2016). Historically, studies on the Dutch famine of 1944 indicated that poor maternal nutrition during pregnancy was associated with low birth weight as well as a greater risk of metabolic and cardiovascular diseases in offspring (Painter et al., 2005). Those studies also showed that maternal nutritional conditions resulted in changes in offspring in DNA methylation patterns in the promoters of genes related to metabolic diseases and cardiac diseases (Tobi et al., 2009), suggesting the involvement of epigenetic modifications in germ cells in inter-/trans-generational influences of the maternal environment. In a rodent model, a maternal nutritional change induced increased DNA methylation of CpG sites in the *A^vy^* allele and inhibition of expression of the corresponding gene, which in turn may result in changes in the hair color of offspring (Waterland and Jirtle, 2003).

With regard to paternal influences, various environmental factors also affect traits of offspring in human as well as rodents (Pembrey et al., 2006; Ng et al., 2010). For instance, studies of paternal inflammatory, nutritional and aging modifications suggested that the environmentally induced epigenetic alterations in sperm can be inherited by the next generation in rodents. Specifically, chemically induced liver damage has been shown to influence paternally inherited suppressive adaptation for liver fibrosis in F1 and F2 generations; these effects are mediated by epigenetic changes in sperm, and up- and downregulation of anti- and pro-fibrogenic genes, respectively, in offspring (Zeybel et al., 2012). Concerning nutritional influences, paternal prediabetes has been shown to alter DNA methylation of sperm genes encoding components of the insulin signaling pathway, leading to glucose intolerance and insulin resistance in the offspring (Wei et al., 2014). Another example is that offspring of males fed a low-protein diet (LPD) exhibit upregulation of genes involved in lipid and cholesterol biosynthesis and decreased levels of cholesterol esters in the liver; these offspring also exhibit changes in the DNA methylation of genes involved in the regulation of lipid metabolism (Carone et al., 2010). In addition, LPD-induced epigenetic changes (such as histone H3 lysine [K] 9 methylation levels and small RNA expression) in testicular germ cells have been shown to be mediated by cyclic AMP-dependent transcription factor 7 (ATF7), with resulting effects on gene expression in offspring (Yoshida et al., 2020). Aging also results in DNA methylation changes of specific genes including neuronal development-related genes in sperm (Yoshizaki et al., 2021).

As in mammals, transgenerational inheritance of the effects of environmental factors via epigenetic modifications has been demonstrated in model organisms such as *Drosophila* and *Caenorhabditis elegans*. Heat shock or osmotic stress in adult or embryonic *Drosophila* induces phosphorylation of *Drosophila* activating transcriptional factor-2 (dATF-2) and results in the release of this protein from heterochromatin; this heterochromatic disruption is an epigenetic event that is transmitted to the next generation in a non-Mendelian fashion (Seong et al., 2011). In *C. elegans*, starvation-induced developmental arrest leads to production of small RNAs that are inherited through at least three consecutive generations; these small RNAs target genes with roles in nutrition (Rechavi et al., 2014).

Transgenerational defects in spermatogenesis have been demonstrated in rodents after maternal exposure to several chemicals, including vinclozolin (Anway et al., 2005), arsenic (Yin et al., 2021), and p,p′-dichlorodiphenoxydichloroethylene (p,p′-DDE), one of the primary metabolic products of the classical organochlorine pesticide, dichlorodiphenoxytrichloroethane (DDT) (Song et al., 2014). Recent work has demonstrated changes of DNA methylation in fetal germ cells and spermatogenetic cells in postnatal testis after prenatal exposure to vinclozolin (Skinner et al., 2019) and DDT (Ben Maamar et al., 2019), but a causal relationship between the altered DNA methylation/gene expression and the spermatogenetic defects has not been determined.

Di(2-ethylhexyl) phthalate (DEHP) is a plasticizer that is used in a wide range of consumer products, such as food packing, medical devices, and wallpapers. Maternal exposure to DEHP has been shown to affect spermatogenesis in male offspring of mouse (Doyle et al., 2013) as well as several other body systems over multiple generations, including the female reproductive system (Brehm et al., 2018), liver (Wen et al., 2020), and anxiety-like behavior (Quinnies et al., 2015). Concerning influences on spermatogenic cells, offspring that are exposed prenatally to DEHP show defects in spermatogenesis, decreases in sperm number, and reduction of sperm motility (Doyle et al., 2013; Prados et al., 2015; Stenz et al., 2017), and F1 sperm demonstrate altered DNA methylation and expression of the genes encoding seminal vesicle secretory protein 2 (Svs2; also known as semenogelin-1); Svs3b; Svs4; and seminal vesicle antigen (Sva), all of which are involved in sperm motility (Stenz et al., 2017). However, possible causes of inter- or trans-generational spermatogenic defects, including epi-mutations of spermatogenesis-related genes, have not been identified; such causes are expected to be essential to understanding the mechanism(s) of the transgenerational effects of DEHP on spermatogenesis. In the present study, we examined changes in DNA methylation and gene expression in fetal male germ cells shortly after maternal DEHP exposure and in spermatogenic cells in adult offspring. We identified candidate epi-mutated genes that are involved in abnormal spermatogenesis in mouse.

## Results

### Defects in spermatogenesis following maternal DEHP exposure

**Figure 1.**
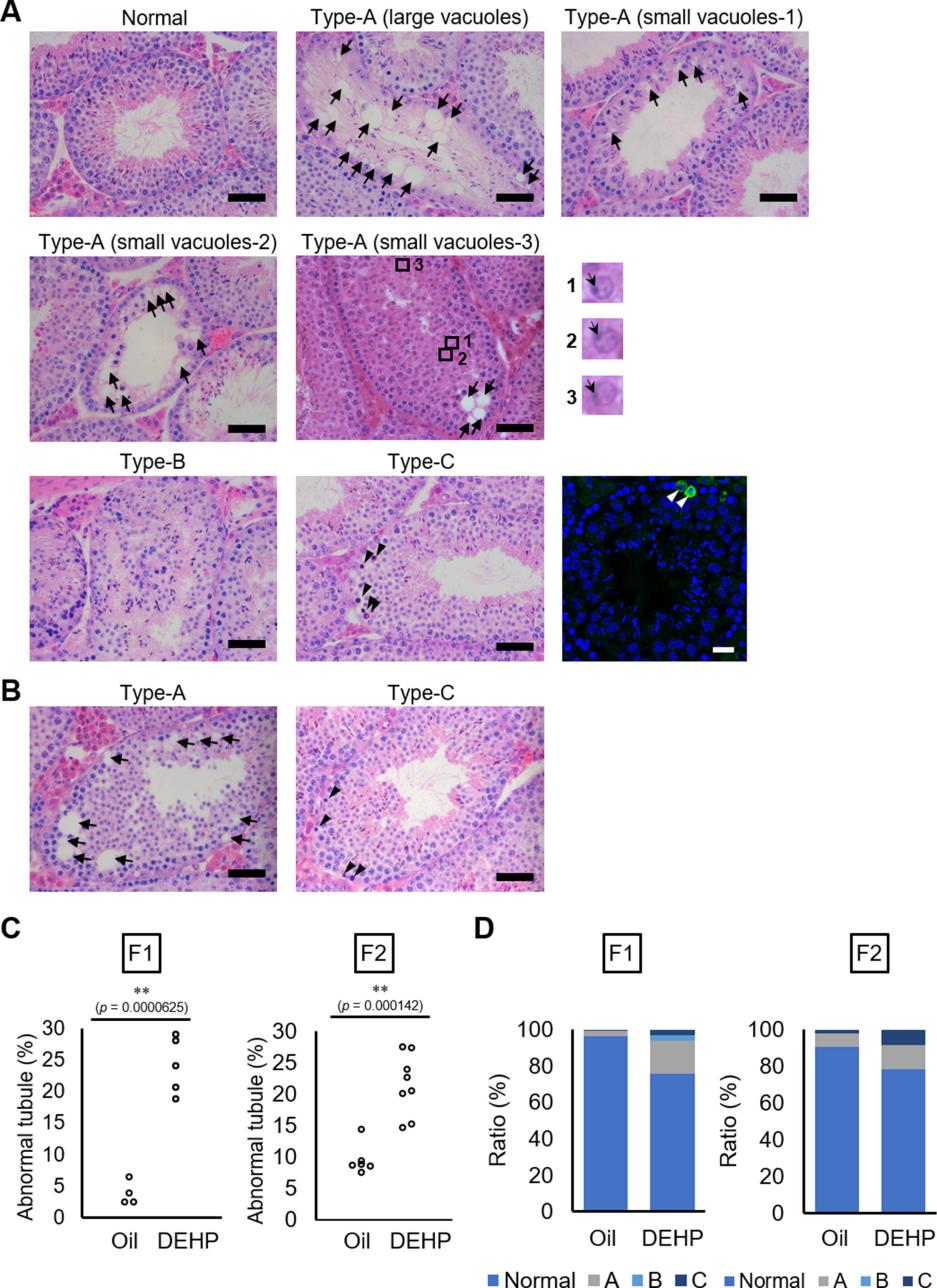
Histological analysis of testicular tubules of C57BL/6 offspring after prenatal di(2-ethylhexyl) phthalate (DEHP) exposure. (**A, B**) Representative images of testicular tubules in F1 (**A**) and F2 (**B**). Abnormal testicular tubules were categorized as ‘Type A’ (presence of various size of vacuoles; indicated by arrows), ‘Type B’ (no lumen and complete loss of germ cell organization), or ‘Type C’ (presence of dead cells; indicated by arrowheads). Type A was further classified into three subtypes as described in Results. Enlarged images corresponding to the rectangular areas in Type A (small vacuoles-3) are shown. Arrows indicate nuclei. Active caspase-3 immunoreactive apoptotic cells (green) are indicated by arrowheads in a picture on the right of the Type C picture. (**C**) Ratios of abnormal tubules in F1 and F2 animals (F1 oil as vehicle control: n = 4; F1 DEHP: n = 5; F2 oil: n = 6, F2 DEHP: n = 8). (**D**) Ratios of abnormal tubule types in F1 and F2. **p<0.01 (unpaired two-sided Student's t-test). Scale bars: 50 μm. Figure 1—source data 1.Numerical source data of abnormal testicular tubule rates (Figure 1C) and ratios of abnormal tubule types (Figure 1D), and data for Kolmogorov–Smirnov test for Figure 1C.

Using C57BL/6 mice, we confirmed the previously reported effects of maternal DEHP exposure on testicular germ cells in embryos and in testes in subsequent generations. First, we used histological evaluation to examine spermatogenesis in the F1 of dams exposed to DEHP and in the F2 resulting from mating of these maternally exposed F1 males with untreated C57BL/6 females. Testicular abnormalities, including vacuoles in tubules (Type A), no lumen in tubules (Type B), and tubules containing apoptotic cells detected as active caspase-3 immunoreactive cells in addition to by histological examination (Type C), were observed in F1 males of the DEHP group, while few abnormal tubules were found in the oil group as vehicle control (Figure 1A), as previously reported (Doyle et al., 2013). In Type A tubules, the size and number of vacuoles varied among tubules. Tubules without germ cells contained large vacuoles. In tubules with less spermatocytes and spermatids and no spermatozoa (small vacuoles-1), with arrested spermatogenesis at the spermatocyte stage (small vacuoles-2), and with abnormal spermatids with peripherally positioned nuclei (enlarged images in Figure 1A) (small vacuoles-3) contained smaller vacuoles (Figure 1A). F2 males of the DEHP group showed similar histological abnormalities (Figure 1B).

**Figure 1—figure supplement 1.**
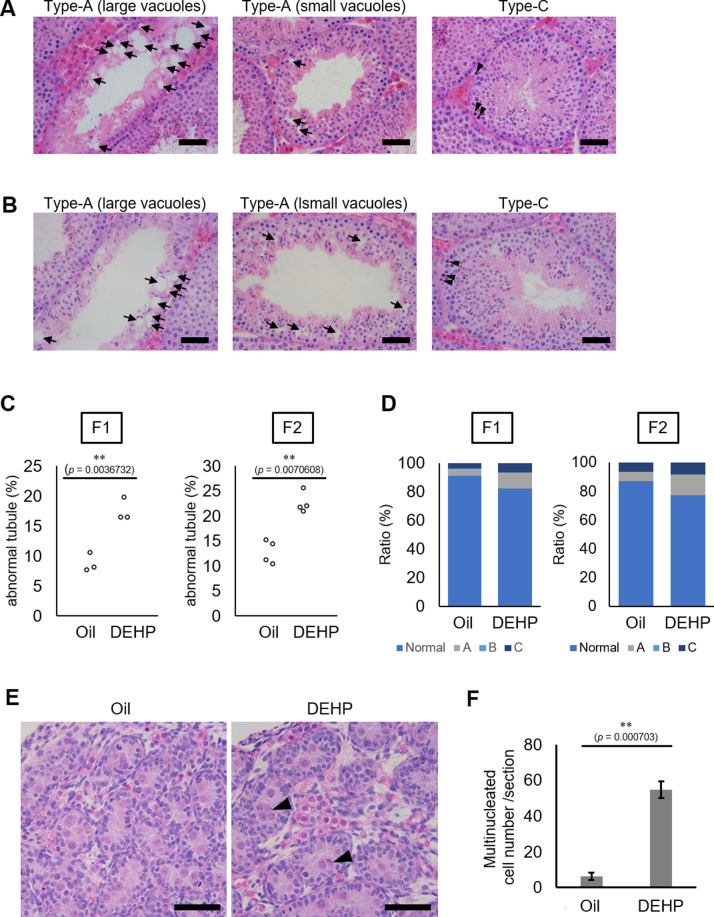
Histological analysis of the testicular tubules of prenatally di(2-ethylhexyl) phthalate (DEHP)-exposed offspring from C57BL/6 females mated with Oct4-deltaPE-GFP transgenic males. (**A, B**) Representative images of testicular tubules in F1 (**A**) and F2 (**B**). Abnormal testicular tubules were categorized as ‘Type A’ (presence of various size of vacuoles; indicated by arrows) and ‘Type C’ (presence of dead cells; indicated by arrowheads). ‘Type B’ (no lumen and complete loss of germ cell organization) abnormal tubules were very rare in F1 and absent in F2. Arrowheads indicate apoptotic cells. (**C**) Ratios of abnormal tubules in F1 and F2 testes (F1: n = 3; F2: n = 4). (**D**) Ratios of abnormal tubule types in F1 and F2. (**E**) Representative histology of testis from oil- or DEHP-treated E19.5 embryos. Multinucleated cells are indicated by arrowheads. (**F**) Quantification of the multinucleated cell number per section. Values are plotted as mean ± SEM of all serial sections from one testis in each of three independent embryos. **p<0.01 (unpaired two-sided Student's t-test). Scale bars: 50 μm. Figure 1—figure supplement 1—source data 1.Numerical source data of abnormal testicular tubule rates (Figure 1—figure supplement 1C), ratios of abnormal tubule types (Figure 1—figure supplement 1D), and multinucleated cells (Figure 1—figure supplement 1F), and data for Kolmogorov–Smirnov test for Figure 1—figure supplement 1C, F.

Quantitative evaluation of the abnormal testicular tubules showed six- and twofold increases of the ratios of the abnormal tubules in the DEHP-exposed F1 and F2 testes, respectively, compared with those in the oil-exposed groups (Figure 1C, Figure 1—source data 1). Classification of the types of the abnormalities, as shown in Figure 1A, revealed that Type B was not observed in F2 (Figure 1D, Figure 1—source data 1), suggesting some difference in the nature of spermatogenic failure between the F1 and the F2 testes. We also observed similar testicular abnormalities in F1 and F2 males derived from Oct4-deltaPE-GFP transgenic mice with a C57BL/6 background, which were used for isolating fetal testicular germ cells (Figure 1—figure supplement 1A–D, Figure 1—figure supplement 1—source data 1). In addition, a significant increase in the number of multinucleated germ cells was observed in embryonic day (E) 19.5 testis in animals prenatally exposed to DEHP compared with those in an oil-exposed group (Figure 1—figure supplement 1E, F, Figure 1—figure supplement 1—source data 1), as reported previously (Ungewitter et al., 2017). These results indicated that our maternal DEHP exposure experiments reproduced the spectrum of testicular abnormalities described in a previous report (Doyle et al., 2013).

### Changes of DNA methylation in fetal and adult testicular germ cell populations of F1 following maternal DEHP exposure

**Figure 2.**
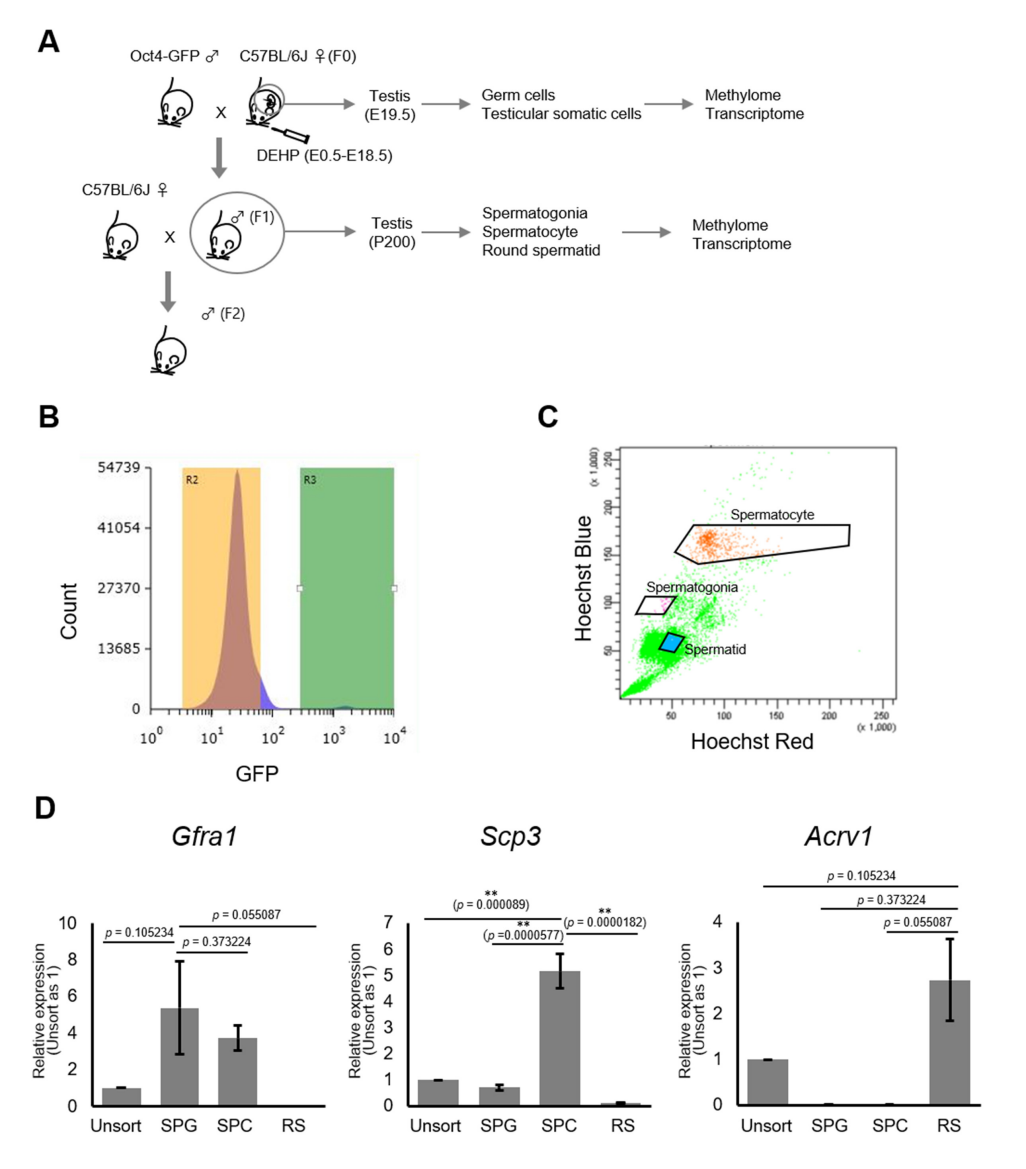
Purification of germ cells from E19.5 and adult testis. (**A**) The bleeding schema of the experiments. (**B**) A representative FACS histogram of E19.5 testis. GFP-positive germ cells in the R3 gate (green color), and GFP-negative testicular somatic cells in the R2 gate (yellow color), were collected. (**C**) A representative FACS plot of F1 testicular cells stained with Hoechst 33342. Gates for spermatogonia, spermatocytes, and round spermatids are indicated. (**D**) Relative expression of stage-specific germ cell marker genes in F1 sorted cells was determined by RT-qPCR and consisted of the following: *Gfra1* for spermatogonia, *Scp3* for spermatocytes, and *Acrv1* for spermatids. SPG: spermatogonia; SPC: spermatocytes; RS: round spermatids. Values were plotted as mean ± SEM of each cell sample from six individuals of F1. Statistics for data in (**D**) was unpaired two-sided Student's t-test. Figure 2—source data 1.Numerical source data for relative gene expression of *Gfra1*, *Scp1*, and *Acrv1*, and data for Kolmogorov–Smirnov test for Figure 2D.SPG: spermatogonia; SPC: spermatocytes; RS: round spermatids.

**Figure 3.**
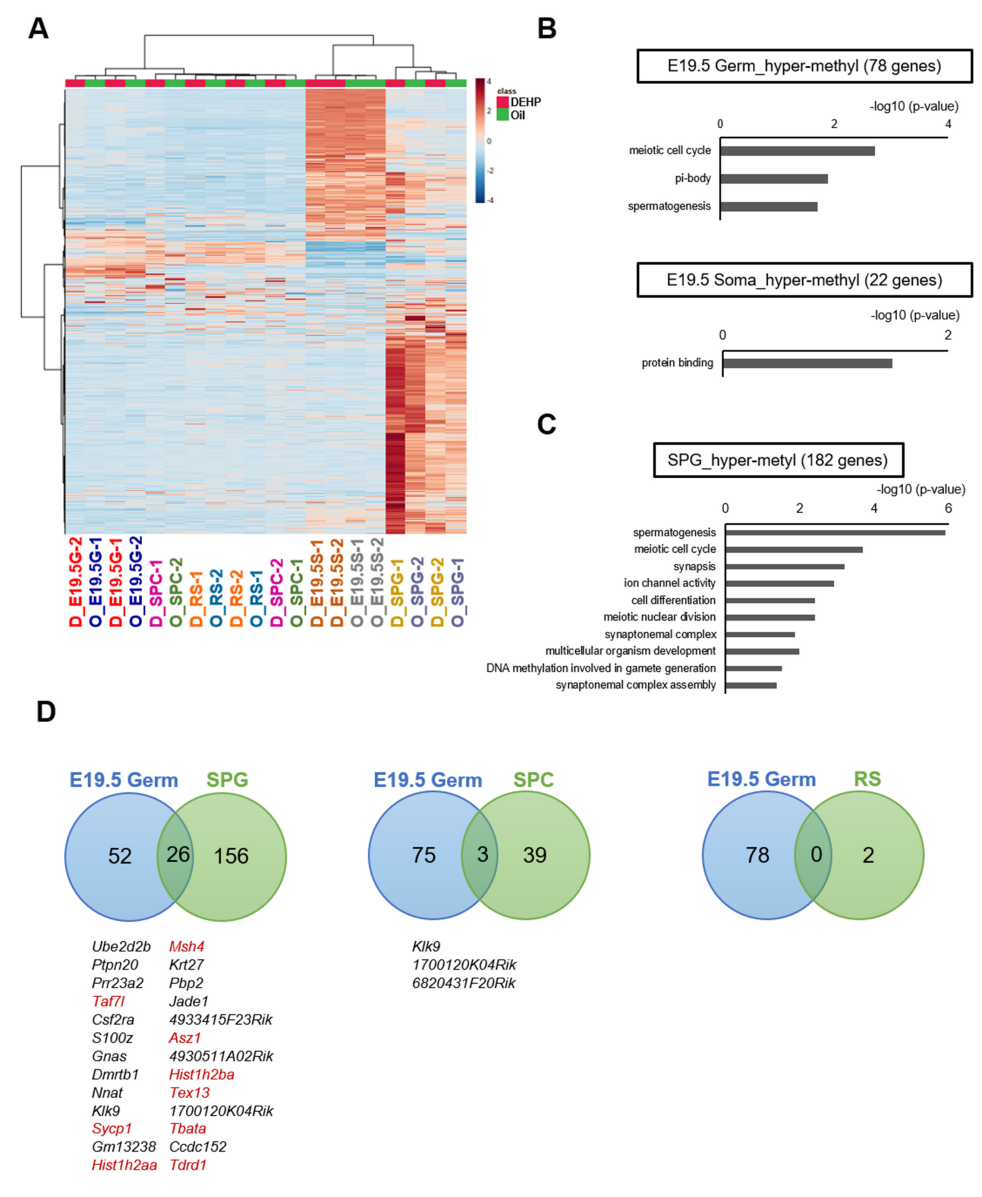
Methylome of testicular cells of F1 after maternal exposure to di(2-ethylhexyl) phthalate (DEHP). (**A**) Heatmap of promoter DNA methylation levels in the testicular cell populations prenatally exposed to oil as vehicle control (O) or DEHP (D) (clustering distance: Euclidean; clustering method: Ward). Each colored cell on the map corresponds to normalized value of methylation percentage. Reddish and bluish colors represent relatively hyper- and hypomethylation, respectively. E19.5G: E19.5 germ cell; E19.5S: E19.5 testicular somatic cells; SPG: spermatogonia; SPC: spermatocytes. (**B, C**) Functional annotation chart of hypermethylated (more than 5% increase in DEHP-treated samples compared to oil-treated samples) genes in E19.5 germ cells (Germ) and testicular somatic cells (Soma) (**B**) and in spermatogonia (**C**) using DAVID. Statistically significant (p<0.05) GO terms with BH-corrected p-values are shown. Threshold was FDR < 0.05. (**D**) Venn diagram analysis of the hypermethylated genes in E19.5 germ cells, SPG, SPC, or round spermatids (RS). Genes that are hypermethylated in E19.5 germ cell and SPG or SPC are listed, and spermatogenesis-related genes are shown in red. Figure 3—source data 1.Analytical codes of reduced representation bisulfite sequencing for Figure 3.

To determine DNA methylation changes in germ cells following maternal DEHP exposure, we performed reduced representation bisulfite sequencing (RRBS) of DNA from germ cells purified from E19.5 testes and from adult F1 testes at approximately postnatal day (P) 200 (Figure 2A). Germ cells and gonadal somatic cells in E19.5 testes were purified as Oct4-deltaPE-GFP-positive and negative cells, respectively, by flow cytometry (Figure 2B). Spermatogonia, spermatocytes, and round spermatids were purified from adult testes by flow cytometry based on their profiles when stained with Hoechst dye, and enrichment of each cell type was confirmed by screening for the expression of known marker genes (*Gfra1* for spermatogonia, *Scp3* for spermatocytes, and *Acrv1* for round spermatids; Figure 2C, D, Figure 2—source data 1). Relatively high *Gfra1* expression in the spermatocyte fraction may be due to contaminated spermatogonia in the spermatocyte gate. Comparison of methylation levels in CpG cytosines in various genomic features (including promoters, gene bodies, CpG islands, CpG island shores, and transposons) showed similar methylation levels between the oil- and the DEHP-exposed groups in each germ cell population in all genomic regions (Supplementary file 1). Heatmap analysis also showed moderate differences in promoter methylation profiles between the oil and the DEHP groups (Figure 3A, Figure 3—source data 1). At the same time, the methylation profiles differed strikingly between spermatogonia and other germ cell populations, such that the promoters in spermatogonial DNA tended to be hypermethylated compared to those in other cell populations, a result that is consistent with the mean methylation levels shown in Supplementary file 1.

**Figure 3—figure supplement 1.**
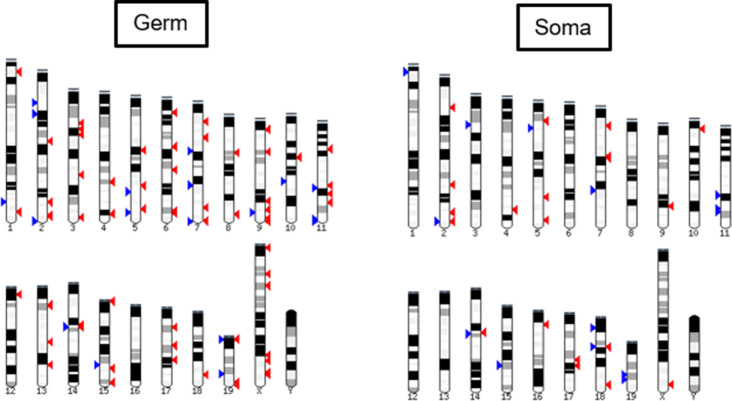
Chromosomal positions of differentially promoter-methylated genes in E19.5 germ and testicular somatic cells. Red arrowhead: more than 5% increase in methylation in the di(2-ethylhexyl) phthalate (DEHP) group compared to the oil group; blue arrowhead: more than 5% decrease in methylation in the DEHP group compared to the oil group.

We next attempted to identify differentially methylated regions (DMRs) in the DNA of E19.5 germ cells when comparing between the oil and DEHP groups. This analysis identified 80 and 22 promoters (78 and 20 genes) with hyper- and hypomethylation, respectively, in DEHP groups (compared to oil groups; Supplementary file 2). These DMRs were widely distributed across the genome in both germ cells and testicular somatic cells (Figure 3—figure supplement 1). Functional enrichment analysis revealed that spermatogenesis-related gene ontology (GO) terms such as meiotic cell cycle and spermatogenesis were enriched in hypermethylated genes in germ cells, while such enrichment was not observed in hypermethylated genes in testicular somatic cells (Figure 3B, Supplementary file 2). In addition, specific functional terms were not enriched in the hypomethylated genes shown in Supplementary file 2.

We also compared methylation in F1 adult testicular germ cells and identified 191, 43, and 2 promoter regions (182, 42, 2 genes) with hypermethylation in spermatogonia, spermatocytes, and round spermatids, respectively, in the DEHP group compared with those in the oil group, among which spermatogenesis-related genes were enriched only in spermatogonia (Figure 3C, Supplementary file 3). GO term enrichment with false discovary rate (FDR) < 0.05 was not found among hypermethylated genes in spermatocytes and round spermatids. As in E19.5 germ cells, only a small number of hypomethylated genes were identified, and functional GO terms were not enriched for these genes (Figure 3B, Supplementary file 3).

We then examined whether the hypermethylated promoter regions detected in the E19.5 germ cells were maintained in F1 testicular germ cells (Figure 3D). We found 26 genes that were hypermethylated in both E19.5 germ cells and F1 spermatogonia; of these 26 genes, 9 encoded products related to spermatogenesis. Meanwhile, no hypermethylated and spermatogenesis-related genes were found to be shared between the E19.5 germ cells and spermatocytes or round spermatids (Figure 3D). These results implied that the DEHP preferentially induced hypermethylation in spermatogenesis-related genes in fetal germ cells, which is maintained up to the spermatogonial stage in F1 testis, though this methylome analysis has low statistical power to capture the reliable global differences of DNA methylation due to the small number of replicates.

### Changes in the expression of the hypermethylated spermatogenesis-related genes in F1 spermatogonia

We next examined changes in gene expression in E19.5 and F1 testicular germ cells following maternal DEHP exposure. Overall, gene expression profiles obtained by RNA-seq showed no remarkable differences between the oil and DEHP groups in each germ cell population (Figure 4—figure supplement 1A, Figure 4—figure supplement 1—source data 1). GO analysis of the differentially expressed genes (DEGs) between the oil and the DEHP groups showed enrichment of several biological processes in F1 testicular germ cell populations (Figure 4—figure supplement 1B, Supplementary file 4). Enrichment of sperm motility-related GO terms such as cilium and cilium movement was observed in upregulated genes in spermatogonia and spermatocytes, although meiosis or spermatogenesis-related genes were not enriched in the downregulated genes. In addition, global changes of gene expression captured by the RNA-seq analysis shown here are not fully reliable because of minimum biological replicates.

**Figure 4.**
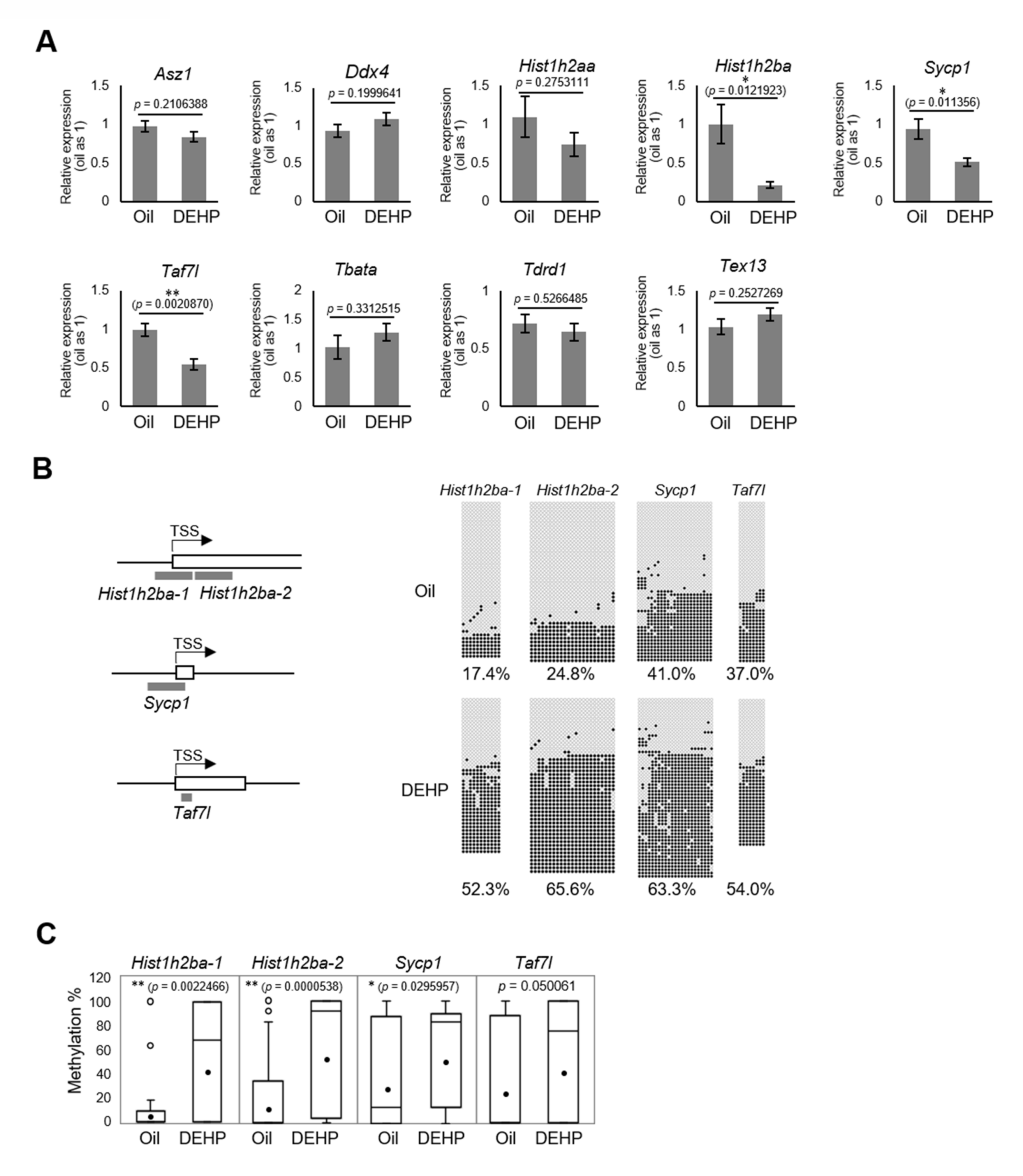
Gene expression and promoter methylation of spermatogenesis-related genes hypermethylated in both E19.5 germ cells and F1 spermatogonia following prenatal di(2-ethylhexyl) phthalate (DEHP) exposure. (**A**) Relative mRNA levels in prenatally oil- or DEHP-treated adult F1 spermatogonia, as determined by RT-qPCR. Values of oil group are defined as 1.0. Values are plotted as mean ± SEM for samples obtained from six individuals. (**B**) The regions detected in bisulfite sequencing of *Hist1h2ba*, *Sycp1*, and *Taf7*l are indicated in gray bars in the left panel. TSS: transcription start site. Boxes indicate the first exon. Methylation status of these regions in F1 spermatogonia obtained from four individuals is indicated in the right panel. Methylated and unmethylated CpGs are presented as closed circles and open circles, respectively. The percentage of methylated CpGs is indicated. (**C**) Box-whisker plots of the CpG methylation levels shown in (**B**). The lines inside the boxes show the medians. The whiskers indicate the minimum and maximum values. Open and closed circles indicate outliers and mean value, respectively. *p<0.05, **p<0.01 (unpaired two-sided Student's t-test in **A**, Mann–Whitney U test in **C**). Figure 4—source data 1.Numerical source data for relative gene expression (Figure 4A) and DNA methylation status (Figure 4D), and data for Kolmogorov–Smirnov test for Figure 4A.

We then focused on the nine candidate spermatogenesis-related genes that were hypermethylated in both E19.5 germ cells and F1 spermatogonia in the RRBS analysis (Figure 3D) and individually evaluated their expression and methylation levels; specifically, we used reverse transcription quantitative polymerase chain reaction (RT-qPCR) to examine the expression of these genes in F1 spermatogonia. This assay showed that the expression of *Hist1h2ba*, *Sycp1*, and *Taf7l* was significantly decreased in the DEHP group (compared to the oil group; Figure 4A, Figure 4—source data 1). We also examined the methylation levels of the promoter regions for *Hist1h2ba*, *Sycp1*, and *Taf7l* by bisulfite sequencing. As shown in Figure 4B, C, Figure 4—source data 1, methylation of the CpG sites in two regions in the *Hist1h2ba* promoter and one region in *Sycp1* promoter was significantly elevated in DEHP-exposed spermatogonia (compared to the levels in the oil group). *Taf7l* in DEHP-exposed spermatogonia also tended to be hypermethylated, though this effect was not statistically significant (Figure 4, Figure 4—source data 1). These results suggested that hypermethylation of *Hist1h2ba*, *Sycp1*, and *Taf7l* in F1 spermatogonia following maternal DEHP exposure could contribute to the decreased expression of these genes, leading in turn to spermatogenic abnormalities.

**Figure 4—figure supplement 1.**
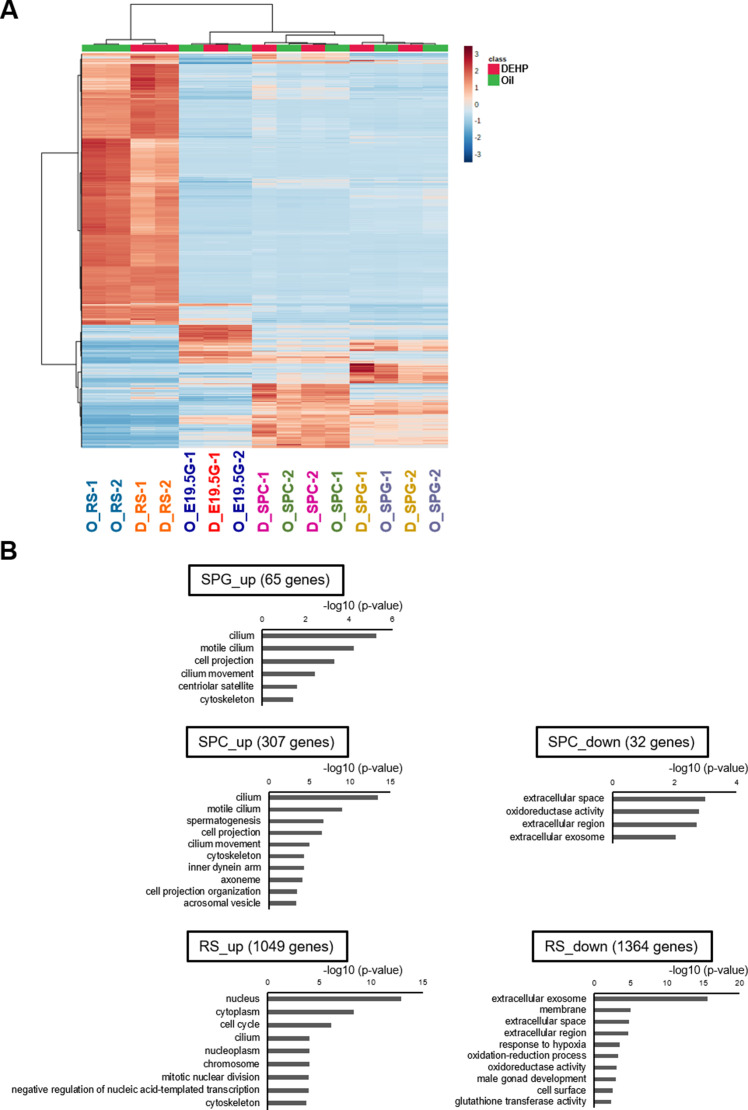
Transcriptome of testicular germ cells after prenatal di(2-ethylhexyl) phthalate (DEHP) exposure. (**A**) A heatmap of TMM-normalized RNA-seq read count values in E19.5 and adult germ cell populations of F1 prenatally exposed to oil (O) or DEHP (D) (clustering distance: Euclidean; clustering method: Ward). Each colored cell on the map corresponds to normalized value of TMM-normalized read count. Reddish and bluish colors represent relatively up- and downregulation, respectively. (**B**) Functional annotation chart of genes exhibiting up- or downregulation (log2-fold-change > 1 or <−1, FDR < 0.05) following DEHP exposure (compared to oil exposure) in E19.5 germ cells (**B**) and F1 germ cell populations. Statistically significant (p<0.05) GO terms with BH-corrected p-values are shown. Threshold was FDR < 0.05. SPG: spermatogonia; SPC: spermatocytes; RS: round spermatids. Figure 4—figure supplement 1—source data 1.Analytical codes of RNA-seq for Figure 4—figure supplement 1.

**Figure 4—figure supplement 2.**
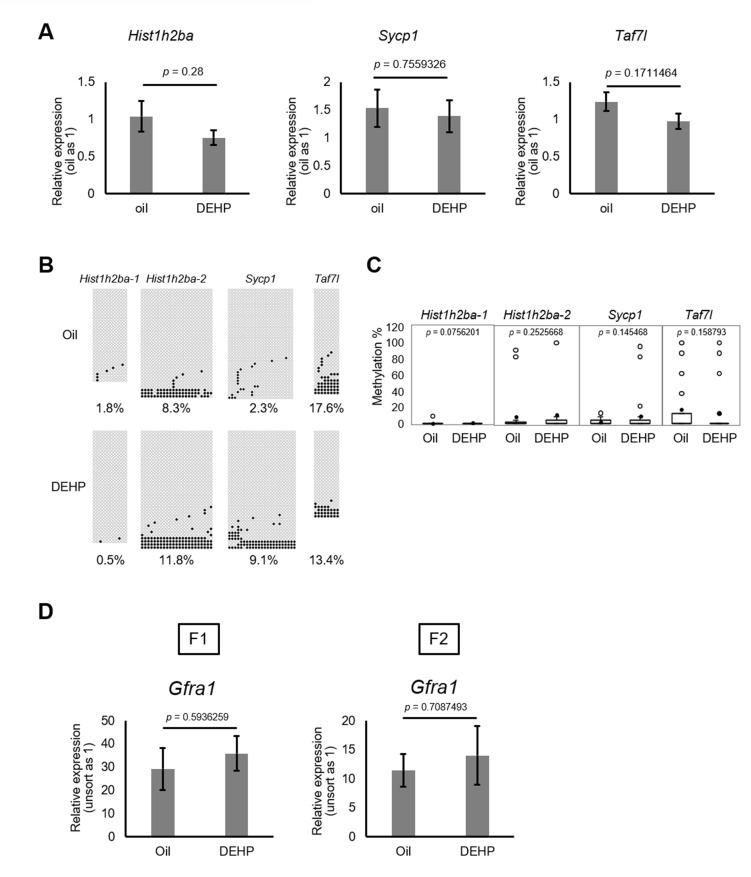
Gene expression and promoter methylation of *Hist1h2ba*, *Sycp1*, and *Taf7l* in F2 spermatogonia. (**A**) Relative mRNA levels determined by RT-qPCR in prenatal oil- or di(2-ethylhexyl) phthalate (DEHP)-treated adult F2 spermatogonia. Values are plotted as mean ± SEM of spermatogonia samples obtained from three individuals. (**B**) Methylation status of these regions determined by bisulfite sequencing in spermatogonia obtained from three individuals. Methylated and unmethylated CpGs are presented as closed circles and open circles, respectively. The percentage of methylated CpGs is indicated. (**C**) Box-whisker plots of the CpG methylation levels shown in (**B**). The lines inside the boxes show the medians. The whiskers indicate the minimum and maximum values. Open and closed circles indicate outliers and mean value, respectively. (**D**) Relative expression of *Gfra1* in F1 and F2 sorted spermatogonia determined by RT-qPCR. Values were plotted as mean ± SEM of each cell sample from four individuals of F1 and three individuals of F2. Statistics for data in (**A**) and (**D**) was unpaired two-sided Student's t-test and that for (**C** )was Mann–Whitney U test. Figure 4—figure supplement 2—source data 1.Numerical source data for relative gene expression Figure 4—figure supplement 2A, D and DNA methylation status (Figure 4—figure supplement 2C), and data for Kolmogorov–Smirnov test for Figure 4—figure supplement 2A, D. SPG: spermatogonia.

**Figure 5.**
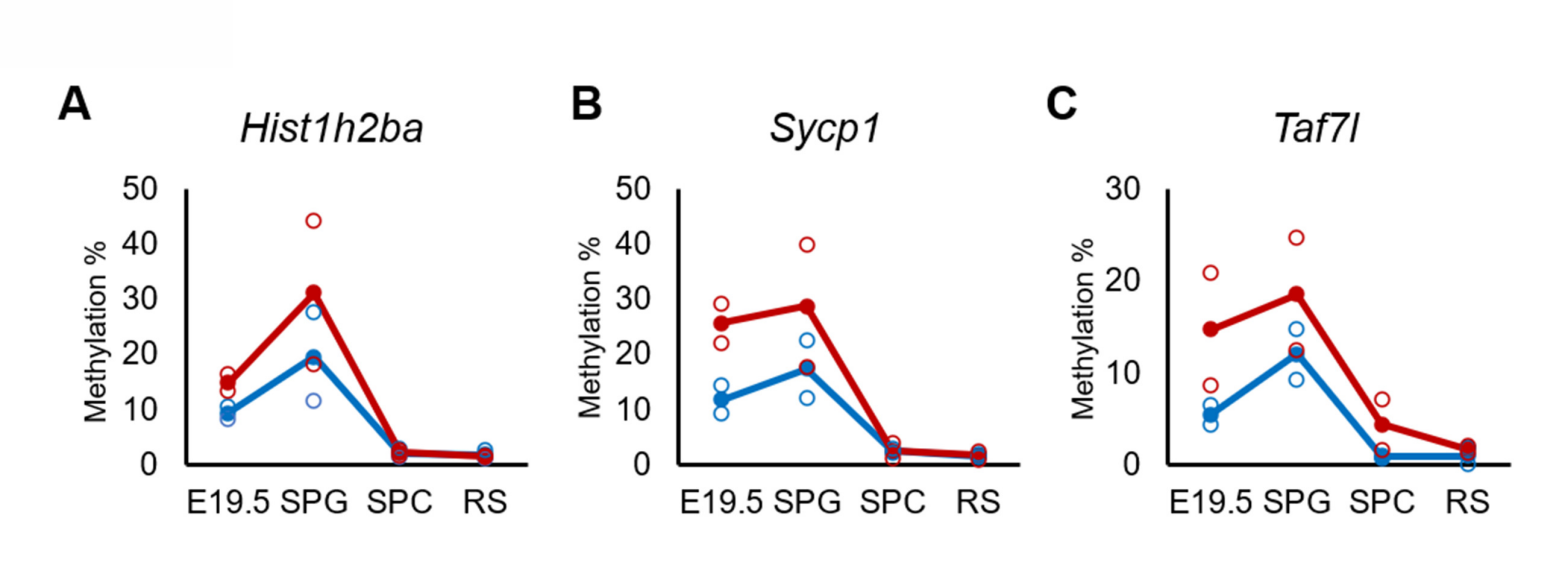
Changes in DNA methylation of the candidate epi-mutated genes in di(2-ethylhexyl) phthalate (DEHP) (red) and oil (blue)-treated E19.5 and adult F1 germ cell populations. (**A–C**) Values of promoter methylation of each replicate (reduced representation bisulfite sequencing [RRBS], n = 2) of *Hist1h2ba* (**A**), *Sycp1* (**B**), and *Taf7l* (**C**) are plotted by open red or blue circles, and means of two replicates are shown by closed circles. Figure 5—source data 1.Numerical source data of DNA methylation level for Figure 5A–C.SPG: spermatogonia; SPC: spermatocytes; RS: round spermatids.

Defects in spermatogenesis following maternal DEHP exposure also were observed in F2 testis. Therefore, we considered it likely that the hypermethylation of the spermatogenesis-related genes is maintained throughout spermatogenesis to transmit epigenetic traits to the F2 generation. However, none or few hypermethylated genes in E19.5 germ cells were shared between spermatocytes and round spermatids in F1 testis (Figure 3D), and methylation levels of the above-mentioned three spermatogenesis-related genes (*Hist1h2ba*, *Sycp1*, and *Taf7l*) in spermatogonia were decreased along with spermatogenesis (Figure 5, Figure 5—source data 1). In addition, the methylation level in the DEHP group was decreased more drastically than was that in the oil group, achieving levels similar to those observed in round spermatids in the oil group. We also examined the expression and methylation of *Hist1h2ba*, *Sycp1*, and *Taf7l* in F2 spermatogonia. We found that F2 spermatogonia were not significantly altered in these parameters, while they exhibited a weak and statistically not significant tendency to decreased expression of *Hist1h2ba*, *Sycp1*, and *Taf7l,* and to hypermethylation of *Hist1h2ba* and *Sycp1* in the DEHP-treated groups compared to the control groups (Figure 4—figure supplement 2A–C, Figure 4—figure supplement 2—source data 1).

**Figure 6.**
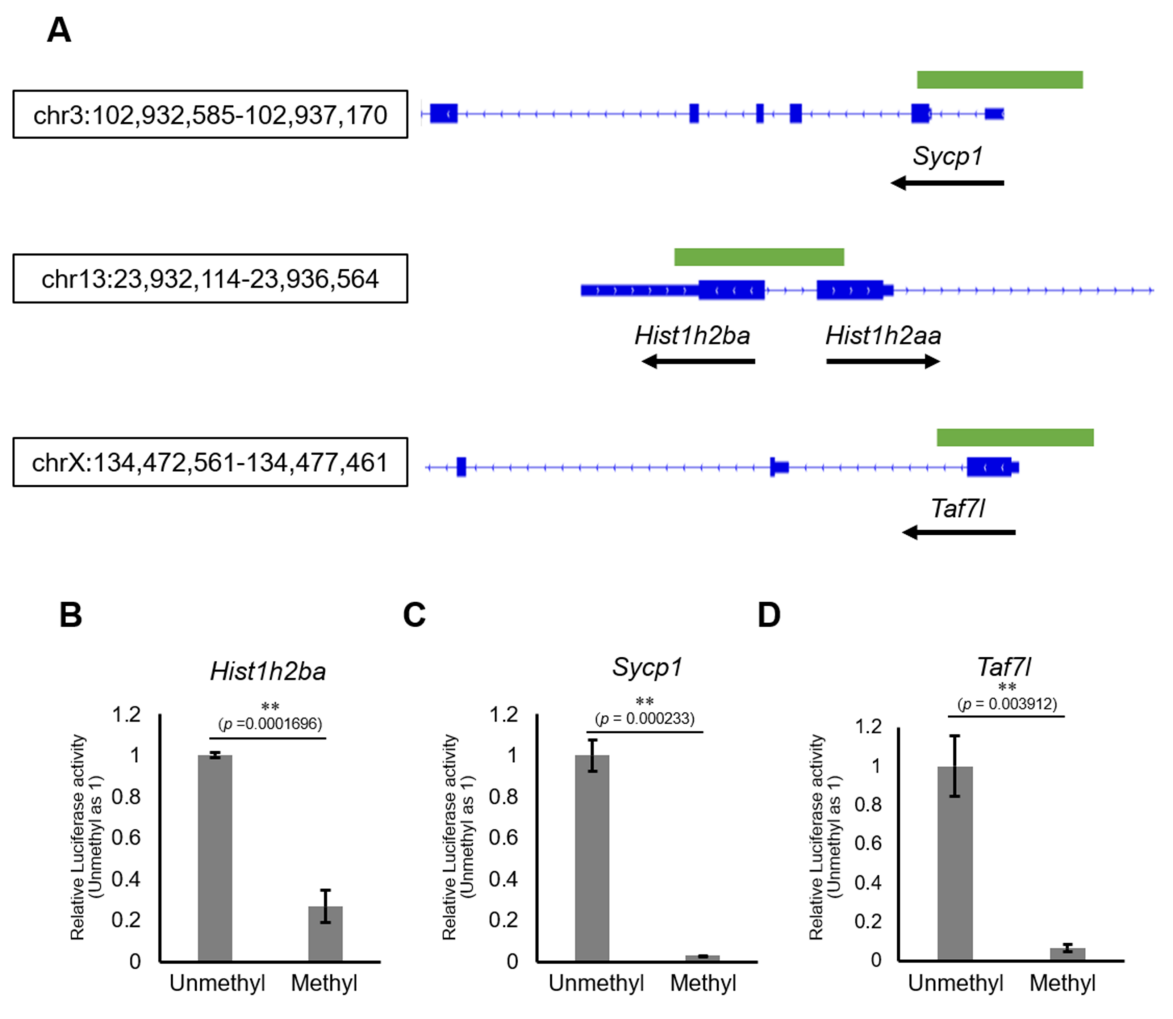
The effects on gene expression of promoter methylation in the candidate epi-mutated loci. (**A**) Gene structures of *Hist1h2ba*, *Sycp1*, and *Taf7l.* Exons are indicated by blue bars, and the promoter regions (defined as ± 500 bp from transcription start site) used for firefly luciferase (Luc) assays are indicated by green bars. (**B**–**D**) The Luc activity of the reporter vector with either methylated or unmethylated promoters of *Hist1h2ba* (**B**), *Sycp1* (**C**), and *Taf7l* (**D**) in HEK293T cells. Luc activity generated by the indicated vector was normalized to that generated by the *Renilla* phRL-TK vector. The Luc activity by the unmethylated vectors was defined as 1.0. Values are plotted as mean ± SEM of three independent experiments (n = 3). **p<0.01 (unpaired two-sided Student's t-test). Figure 6—source data 1.Numerical source data for relative luciferase activity and data for Kolmogorov–Smirnov test for Figure 6B–D.

We next examined the involvement of DNA methylation in regulation of the expression of *Hist1h2ba*, *Scyp1*, and *Taf7l*. Specifically, we employed a luciferase (Luc) reporter assay in cultured cells using promoter regions (−500 bp to +500 bp from the transcription start sites [TSSs]) of each of these genes (Figure 6A). We found that Luc activity was significantly decreased by methylation of the promoter of each gene (Figure 6B–D, Figure 6—source data 1). The results supported the hypothesis that hypermethylation of *Hist1h2ba*, *Scyp1*, and *Taf7l* results in decreased expression in testicular germ cells.

## Discussion

### Close correlation between DNA methylation changes and abnormal spermatogenesis after maternal DEHP exposure

Induction of abnormal spermatogenesis in offspring following maternal DEHP exposure was first demonstrated using CD1 mice (Doyle et al., 2013) and subsequently was found in several strains of mice (including C57BL/6) and rats (Prados et al., 2015; Chen et al., 2015). In the present study, we also used C57BL/6 as well as Oct-4-deltaPE-GFP transgenic mice in a C57BL/6 genetic background to purify GFP-expressing fetal germ cells by flow cytometry. We first administered pregnant mice with 300 mg/kg DEHP, which is the same dose as used in a previous study with C57BL/6 (Prados et al., 2015); however, we obtained few pups under those conditions, presumably reflecting embryonic lethality at this dose of DEHP. We then tested different doses of DEHP and identified dose levels at which we could obtain pups that showed germ cell abnormalities in F1 and F2 offspring and in F1 embryos that were similar to those reported previously (Doyle et al., 2013; Stenz et al., 2017; Ungewitter et al., 2017) at 100 or 150 mg/kg DEHP (Figure 1, Figure 1—figure supplement 1, Figure 1—source data 1, Figure 1—figure supplement 1—source data 1). Therefore, our experiments using C57BL/6 and Oct-4-deltaPE-GFP transgenic mice successfully reproduced the previously reported DEHP-induced histological abnormalities in testis, despite the use of a lower DEHP dose.

Using this experimental model, we identified a close correlation between DNA hypermethylation of spermatogenesis-related genes and their downregulation in F1 spermatogonia following maternal DEHP exposure (Figure 4, Figure 4—source data 1). Previous reports have described promoter hypermethylation and downregulation of the *Svs3ab* gene, a gene that is involved in sperm motility, in the sperm of F1 males following maternal DEHP exposure (Stenz et al., 2017; Stenz et al., 2019). Given that maternal DEHP exposure induces not only defective sperm but also abnormal spermatogenesis, we considered it likely that spermatogenesis-related genes are affected by DEHP (Figure 1, Figure 1—figure supplement 1, Figure 1—source data 1, Figure 1—figure supplement 1—source data 1); consistent with this hypothesis, we demonstrated promoter hypermethylation of three spermatogenesis-related genes, *Hist1h2ba*, *Sycp1*, and *Taf7l,* as well as downregulation of the expression of these three genes in the F1 spermatogonia following maternal DEHP exposure (Figure 3, Figure 4, Figure 3—source data 1, Figure 4—source data 1).

We also found that methylation of the promoters of these genes reduced their expression, as assessed by the methyl-luciferase assay (Figure 6, Figure 6—source data 1), a result that implies the involvement of methylation of those genes in their downregulation. In a previous study, hypermethylation of some genes, but not that of spermatogenesis-related genes, was demonstrated in fetal male germ cells following maternal DEHP exposure (Prados et al., 2015); however, that study did not evaluate involvement of the hypermethylated genes in abnormal spermatogenesis. The inconsistency between our results and those of previous studies regarding hypermethylation of spermatogenesis-related genes by DEHP may be due, in part, to the use of different mouse strains. Specifically, our study employed mice with a C57BL/6 genetic background, as described above, while the previous study (which did not demonstrate testicular abnormalities) used mice with FVB and 129S1 genetic backgrounds (Iqbal et al., 2015). In addition, it has been reported that DEHP exposure does not cause any testicular abnormalities in FVB mice (Prados et al., 2015). Given that deficiencies in *Hist1h2ba*, *Sycp1*, or *Taf7l* result in abnormal spermatogenesis (as described below), our results imply that the downregulation of those genes following DEHP exposure is a cause of abnormal spermatogenesis.

### Effects of insufficient gene expression of Hist1h2ba, Scyp1, and Taf7l in spermatogenesis

The two histone variants HIST1H2AA and HIST1H2BA are abundant in the testis (Trostle-Weige et al., 1982), and male mice lacking both *Hist1h2aa* and *Hist1h2ba* are sterile (Shinagawa et al., 2015). Abnormal localization of nuclei in spermatids following maternal DEHP exposure (Figure 1A, Figure 1—source data 1) is consistent with the phenotype observed in animals lacking both *Hist1h2ba* and *Hist1h2aa*, a phenotype that likely reflects impaired replacement of histones by protamine during spermiogenesis (Shinagawa et al., 2015). The *Hist1h2aa* and *Hist1h2ba* genes are located tandemly in inverted orientation on chromosome 13 in mouse (Figure 6), and expression of the two loci is controlled by a shared promoter located between the two genes (Huh et al., 1991). Consistent with that structure, we found a tendency toward reduced *Hist1h2aa* expression in addition to significant downregulation of *Hist1h2ba* expression (Figure 4A, Figure 4—source data 1) following maternal DEHP exposure, suggesting that hypermethylation of the shared promoter affects the expression of these two genes in testicular germ cells.

SYCP1 is a component of the synaptonemal complex, a structure that connects homologous chromosomes in meiotic prophase; *Sycp1* null mice are sterile because of spermatogenic arrest at the pachytene stage (de Vries, 2005). A similar abnormality was observed in F1 mice following maternal DEHP exposure (Figure 1A), a result that suggests that downregulation of *Sycp1* expression is a cause of abnormal spermatogenesis following maternal DEHP exposure. TAF7L is a testis-specific transcription-associated factor (Pointud et al., 2003). *Taf7l* null testis exhibits sperm with abnormal morphology and reduced motility, and large vacuoles are seen in the seminiferous tubules (Cheng et al., 2007); these defects resemble the abnormal tubules seen in the F1 testis following maternal DEHP exposure. Taken together, these observations indicate that the *Hist1h2ba*, *Sycp1*, and *Taf7l* genes are epi-mutated following maternal DEHP exposure; the resulting downregulation of these gene may be a cause of abnormal spermatogenesis.

### Mechanism of hypermethylation of spermatogenesis-related genes in fetal germ cells following maternal DEHP exposure

Our study revealed that the spermatogenesis-related genes were hypermethylated in fetal germ cells (Figure 3, Figure 3—source data 1), suggesting a possible preference of DEHP-induced DNA methylation for germ cells and/or spermatogenesis-related genes. Previous studies have shown that maternal DEHP exposure induces DNA methylation not only in testis but also in other tissues such as adrenal gland and liver (Quinnies et al., 2015; Wen et al., 2020); notably, DEHP exposure resulted in a significant increase in the expression, in the liver of the offspring, of *Dnmt1*, a gene encoding a DNA methyltransferase. These results suggested that upregulation of *Dnmt1* by DEHP plays a role in DNA hypermethylation. Change of expression levels of *Dnmts* after maternal DEHP exposure awaits a further study.

Another possible mechanism is involvement of reactive oxygen species (ROS). ROS may be involved in DEHP-induced DNA methylation, given that mono (2-ethylhexyl) phthalate (MEHP), a hydrolyzed metabolite of DEHP, induces ROS production in adult testicular germ cells (Kasahara et al., 2002), and that ROS directly enhances DNA methylation (Afanas'ev, 2014). In fetal germ cells, the chromatin structure around spermatogenesis-related genes is highly accessible (Li et al., 2018) and may facilitate preferential DNA methylation of spermatogenesis-related genes in fetal germ cells in response to the accumulation of ROS following DEHP exposure. Alternatively, DEHP- or MEHP-binding molecules may be involved in the observed DNA methylation. DEHP and MEHP are ligands of peroxisome proliferator-activated receptors (PPARs) and the constitutive androstane receptor (CAR); these receptors regulate downstream pathways (Corton and Lapinskas, 2005; Eveillard et al., 2009) and may thereby promote DNA methylation via unknown mechanisms, including recruitment of DNA methylation-related molecules to target genes. Those possibilities await further investigation.

### Possible mechanisms of intergenerational inheritance of abnormal spermatogenesis following maternal DEHP exposure

Changes of the expression and methylation of *Hist1h2ba*, *Sycp1*, and *Taf7l* in F2 spermatogonia were not statistically significant after maternal DEHP exposure (Figure 4—figure supplement 2, Figure 4—figure supplement 2—source data 1). In our results, methylation levels in these genes in F2 spermatogonia generally were lower than those in F1 spermatogonia, even in the oil-treated group (Figure 4, Figure 4—figure supplement 2B, C, Figure 4—source data 1, Figure 4—figure supplement 2—source data 1); these differences may be due, at least in part, to differences in the purity of the spermatogonia. We found that enrichment of *Gfra1* expression in sorted spermatogonia fractions was higher in those of F1 than those of F2, though its enrichment between oil- and DEHP-treated groups of F1 and of F2 was comparable (Figure 4—figure supplement 2D, Figure 4—figure supplement 2—source data 1). Because the F2 alleles are inherited from DEHP-exposed males and untreated females, the influence of DEHP-induced epigenetic changes reasonably may become weaker or disappear in the F2 alleles compared with those in the F1 alleles. Additionally, those genes became hypomethylated during spermatogenesis in F1 testis (Figure 5, Figure 5—source data 1), suggesting that the hypermethylation of the genes by DEHP is not directly transmitted to the F2. A recent study suggested that binding of transcription factors (TFs) at CpG sites affects the methylation status of these sites during demethylation in primordial germ cells and affects subsequent re-methylation of these sites; notably, CpG sites not bound by TFs could be re-methylated after demethylation (Kremsky and Corces, 2020). These results imply that *Hist1h2ba*, *Sycp1*, and *Taf7l* are demethylated during spermatogenesis, and subsequently are re-methylated at some developmental time point(s) after fertilization.

In the case of embryonic undernutrition, abnormal DNA hypomethylation in F1 sperm is not inherited in F2 sperm and somatic tissues; however, the transcription levels of several genes near the regions with altered DNA methylation in F1 sperm are dysregulated in F2 somatic tissues (Radford et al., 2014). These results suggest that changes in DNA methylation perturb (indirectly) gene expression patterns in subsequent generations. Modified histones that are bound to specific genomic regions, such as H3K4 trimethylation (me3) bound at CpG-rich promoters and H3K9me3 bound at satellite repeats in the sperm genome, are not replaced by protamine during sperm development (Yamaguchi et al., 2018). Maternal undernutrition, as well as maternal DEHP exposure, may affect such histone modifications in F1 sperm in a DNA methylation status-dependent manner, which in turn may influence phenotypes in the F2. Further work will be required to explore this possibility.

## Materials and methods

**Key resources table:** 

Reagent type (species) or resource	Designation	Source or reference	Identifiers	Additional information
Genetic reagent (*Mus musculus*, males)	Oct4-delta PE-GFP	PMID:10646797		C57BL/6J background
Cell line (*Human*)	HEK293T	ATCC RRID:CVCL_0063	CRL-3216	
Antibody	Anti-Cleaved Caspase-3 (rabbit polyclonal)	CST RRID:AB_2341188	Cat# 9661	(1:400)
Recombinant DNA reagent	pCpGL (plasmid)	PMID:17965610		http://www.ag-rehli.de/materials.html
Commercial assay or kit	EZ DNA Methylation-Gold Kit	Zymo Research	Cat# D5005	
Commercial assay or kit	NEBNext Ultra II RNA Library Prep Kit for Illumina	BioLabs	Cat#E7770S	
Commercial assay or kit	Dual-Luciferase Reporter Assay System	Promega	Cat#E1910	
Chemical compound, drug	DEHP	Sigma	Cat#80030	
Chemical compound, drug	Corn oil	Sigma	Cat#C8267	
Software, algorithm	Bismark	PMID:21493656	v0.10.1	https://www.bioinformatics.babraham.ac.uk/projects/bismark/
Software, algorithm	TopHat2	PMID:23618408	V2.1.0	https://ccb.jhu.edu/software/tophat/index.shtml
Software, algorithm	Bowtie2	PMID:22388286	V2.2.6.0	http://bowtie-bio.sourceforge.net/index.shtml
Software, algorithm	featureCounts	PMID:24227677	Packaged in Subread v1.5.0-p2	http://subread.sourceforge.net/
Software, algorithm	edgeR	PMID:19910308	V3.13	https://bioconductor.org/packages/release/bioc/html/edgeR.html

### Animals and DEHP treatment

C57BL/6J mice were purchased from Japan SLC. Oct4-deltaPE-GFP transgenic mice (Yoshimizu et al., 1999) were maintained in a C57BL/6J genetic background. The mice were maintained and bred in an environmentally controlled and specific pathogen-free facility, the Animal Unit of the Institute of Development, Aging and Cancer (Tohoku University), according to the guidelines for experimental animals defined by the facility. Animal protocols were reviewed and approved by the Tohoku University Animal Studies Committee (approval number: 2019AcA-027-01). Maternal DEHP exposure was performed as described previously (Stenz et al., 2017). Briefly, female C57BL/6J mice (8–12 weeks old) were mated with male C57BL/6J or Oct4 (Pou51f)-deltaPE-GFP transgenic mice; noon on the day of mucus plug observation was defined as E0.5. Prenatal exposure to DEHP was performed by oral (gavage) administration of pregnant female mice, performed once per day from E8 to E18. At each administration, 200 μL of either corn oil (Sigma C8267) (for controls) or 200 μL of DEHP (Sigma 80030) diluted in corn oil to provide a dose of 100 mg/kg/day or 150 mg/kg/day for C57BL/6J or Oct4-deltaPE-GFP males, respectively, was delivered by oral gavage. The treated gestating dams were considered the F0 generation (F0), and the pups born from the F0 dams were considered the F1 generation (F1) offspring. The F2 generation was obtained by breeding males of the F1 of DEHP-treated animals or vehicle control animals with nontreated C57BL/6J females. We analyzed 3–8 mice of each condition as biological replicates to statistically evaluate the effect of DEHP.

### Cell line

HEK293T (ATCC #CRL-3216, RRID:CVCL_0063) was confirmed by STR profiling and was negative for mycoplasma. It was cultured in Dulbecco’s modified Eagle medium (DMEM, Gibco 11965-092) containing fetal bovine serum (FBS) (Biosera FB-1380) at 37°C and 5% CO_2_.

### Purification of germ cells

E19.5 embryos were obtained from DEHP- or corn oil-treated female mice; embryonic testes were isolated, and the albuginea was removed in DMEM (Gibco 11965-092) containing 10% FBS (Biosera FB-1380). Testes then were incubated for 15 min at 37°C in phosphate-buffered saline (PBS) containing 0.5 mg/mL trypsin and 0.2 mg/mL EDTA (Sigma T4174). The cells were dissociated by pipetting, and then were filtered through a 40-µm-pore Cell strainer (BD Falcon 352340). A Bio-Rad S3e cell sorter was used to purify viable germ cells with intense Oct4-deltaPE-GFP expression and testicular somatic cells (Somas) without Oct4-deltaPE-GFP expression. For DNA isolation, one-quarter-volume of sorted cell suspension was centrifuged and stored at −80°C after removal of the supernatant. For RNA isolation, the remainder of the cell suspension was centrifuged, resuspended in buffer RLT (QIAGEN 79216) containing 1% beta-mercaptoethanol, and stored at −80°C. For each cell sorting, we obtained 1–16 embryos. The cells obtained from four independent sorting were combined before DNA or RNA extraction, which yielded suspensions containing approximately 12,000 cells for DNA isolation and 40,000 cells for RNA isolation. We repeated four more sorting and combined the sorted cells to obtain a biological replicate. To obtain totally 101,703 cells for oil- or 108,891 cells for DEHP group of germ cells used for the methylome and transcriptome, 43 and 82 embryos were dissected. Dissociation of testicular cells from adult mice and subsequent staining were carried out as described previously (Bastos et al., 2005; Ota et al., 2019). Testes were dissected from F1 or F2 mice at approximately postnatal day (P) 200. After the albuginea was removed, testes were incubated at 32°C for 25 min in 6 mL of Gey’s Balanced Salt Solution (GBSS; Sigma-Aldrich G9779) containing 1.2 mg/mL of Collagenase Type I (Sigma-Aldrich C0130), and the seminiferous tubules were dissociated. Interstitial cells were removed by filtration with the Cell strainer. Seminiferous tubules retained on the filter were collected and incubated at 32°C for 25 min in GBSS containing 1.2 mg/mL of Collagenase Type I and 5 μg/mL DNase (Roche 11284932). Cell aggregates were sheared gently by 10 rounds of pipetting with a wide orifice plastic transfer pipet and filtered through the Cell strainer to remove cell clumps. Cells were washed with GBSS and then resuspended in GBSS containing 1% FBS. Twenty million cells were diluted in 2 mL of GBSS containing 1% FBS and stained with 5 μg/mL of Hoechst 33342 (Invitrogen H3570) for 1 hr at 32°C. Cells were kept on ice and protected from light until sorting. Before sorting, 0.25 μg/mL of propidium iodide (PI, BD 51-66211E) was added to the stained cells, and the mixture was filtered through the Cell strainer. The cells were sorted using a Becton-Dickinson FACSAria II cell sorter. PI-positive cells reflect dead cells, which were first eliminated by gating, and PI-negative living cells were then further fractionated to spermatogonia, spermatocytes, and round spermatids according to their Hoechst staining patterns. Fluorescence of Hoechst 33342 has two emissions wavelength (450 nm, blue, and 675 nm, red), and the stained cells can be separated by signal intensity for the two wavelengths, which partly reflects DNA content of the cells (Goodell et al., 1996). To avoid interference of GFP with red and blue emissions of Hoechst, compensation of fluorescence signals between GFP and Hoechst dye on the flow cytometer was performed using unstained or GFP-negative testicular cells. Confirmation of the purity of the sorted germ cells was evaluated by assessing the expression of stage-specific germ cell marker genes as follows: *Gfra1* for spermatogonia (Hofmann et al., 2005), *Scp3* for spermatocytes (Lammers et al., 1994), and *Acrv1* for round spermatids (Reddi et al., 1995). We obtained two biological replicates for each cell type in F1 testes for transcriptome and methylome; one was composed of the cells from one individual, and the other was composed of pooled cells from two individuals to secure enough number of cells. Each sample was further divided for transcriptome (approximately 50,000 cells) and methylome (approximately 15,000 cells) analyses. For RT-qPCR and bisulfite sequencing, spermatogonia collected from one individual were used as one biological replicate.

### Histological examination

Testes from E19.5 embryos of the F1 generation and P200 male mice of the F1 and F2 generations were fixed overnight at 4°C in Bouin’s solution with rotation, and then were embedded in paraffin. Five-micrometer-thick serial sections were cut and mounted on 3-aminopropyltriethoxysilane (APS)-coated slides (Matsunami APS-01); the mounted sections were deparaffinized, stained with hematoxylin and eosin, and examined using an optical microscope (Leica). Digital images were obtained using a LAS4.4 (Leica), and abnormal tubules were determined by the histological criteria described in the Results section. To determine the percentage of abnormal tubules, the numbers of abnormal tubules and total tubules were counted in each testicular section. Four sections, each 100 μm apart in a given testis, were counted.

### Immunostaining

DEHP-treated testis were fixed 4% paraformaldehyde for overnight at 4°C and embedded in Optimum Cutting Temperature (O.C.T.) compound (Sakura Finetek 4583). The embedded samples were sectioned using a CM3050S cryomicrotome (Leica) to a thickness of 10 μm. The sections were permeabilized, blocked, incubated with Anti-Cleaved Caspase-3 (CST 9661, RRID:AB_2341188, 1:400 dilution) for overnight at 4°C, and then incubated with Anti-rabbit Alexa 488 (Invitrogen A21206, 1:500 dilution) and 1 μg/mL DAPI for 1 hr at room temperature. The sections were washed after primary and secondary antibody treatments. Samples were mounted using Vectashield (Vector H-1000) and observed under a TCS SP8 confocal laser scanning microscope (Leica).

### Reduced representation bisulfite sequencing

The purified cells described above were suspended in a lysis buffer (0.14 mM β-mercaptoethanol, 0.24 mg/mL Proteinase K, 150 mM NaCl, 10 mM Tris-HCl [pH 8.0], 10 mM EDTA [pH 8.0], and 0.1% SDS) and incubated at 55°C for 2 hr. Genomic DNA then was isolated using phenol/chloroform extraction and ethanol precipitation. RRBS libraries were generated as previously reported (Gu et al., 2011). Briefly, 20 ng of genomic DNA was subjected to MspI digestion (NEB, Beverly, MA), with subsequent end repair/dA-tailing reaction using Klenow Fragment (3'−5' exo-) (NEB M0212S) and ligation with Illumina sequencing adapters using T4 DNA ligase (NEB M0202T). The resulting mixture was electrophoresed in NuSieve 3:1 agarose (Lonza 50091), and fragments sized 150–350 bp were excised and then purified using a MinElute Gel Extraction kit (QIAGEN 28604). The purified fragments were treated with sodium bisulfite using an EZ DNA Methylation-Gold Kit (Zymo Research D5005). Library amplification and indexing were performed with KAPA HiFi HotStart Uracil+ ReadyMix (2×) (Kapa Biosystems KK2801). The PCR amplification was carried out as follows: an initial denaturation at 95°C for 2 min; 13 cycles at 98°C for 20 s, 65°C for 30 s, and 72°C for 30 s; and a final 1 min extension at 72°C. The RRBS libraries were purified using Agencourt AMPure XP (Beckman Coulter A63880), quantified with a Kapa Library Quantification Kit (Kapa Biosystems KK4824), and sequenced on an HiSeq 2500 platform (Illumina) with 100 bp single-end reads using a TruSeq SR Cluster Kit v3-cBot-HS (Illumina GD-401-3001) and TruSeq SBS Kit v3-HS (Illumina FC-401-3001). Sequenced reads were processed using an Illumina standard base-calling pipeline (v1.8.2), and the index and adapter sequences were removed. The first and last four bases were trimmed, and the resulting reads were aligned to Mouse Genome Build 37 (mm10) using Bismark (v0.10.1) (Krueger and Andrews, 2011) with default parameters. The methylation level of each cytosine was calculated using the Bismark methylation extractor. We analyzed only CpG cytosines covered with three reads. Annotations of Refseq genes and repeat sequences were downloaded from the UCSC Genome Browser. Refseq genes encoding microRNAs and small nucleolar RNAs as well as mitochondrial DNA were excluded from our analyses. Promoters were defined as regions 500 bp upstream and downstream from TSSs of Refseq transcripts. Gene bodies were defined as transcribed regions of Refseq transcripts except for promoters. We defined percentages of methylated CpG in all CpG sites in the regions of interest as methylation levels, which was estimated by analyzing promoters containing ≧10 CpG cytosines with sufficient coverage for calculation of the methylation levels. Analytical code is indicated in Figure 3—source data 1. DMRs were defined as regions with more than 5% changes in methylation levels in the DEHP-treated samples compared to the oil-treated samples. We analyzed two biological replicates to show reproducibility.

### RNA sequencing

RNA-seq libraries were prepared from total RNA purified from sorted germ cells with a RNeasy Micro Kit (QIAGEN 74004). The libraries constructed by NEBNext Ultra II RNA Library Prep Kit for Illumina (BioLabs E7770S) were clonally amplified on a flow cell and sequenced on HiSeq2500 (HiSeq Control Software v2.2.58, Illumina) with 51-mer single-end sequences. Image analysis and base calling were performed using Real-Time Analysis Software (v1.18.64, Illumina). For gene expression analysis, reads were mapped to the mouse genome (UCSC mm10 genome assembly and NCBI RefSeq database) using TopHat2 (v2.1.0) (Kim et al., 2013) and Bowtie2 (v2.2.6.0) (Langmead and Salzberg, 2012). FeatureCounts (packaged in Subread v1.5.0-p2) (Liao et al., 2014) was used to calculate count data from BAM files. After trimmed mean of M values (TMM) normalization by edgeR (v3.13) (Robinson et al., 2010) operated in R (v4.1.0), DEGs were extracted with log2-fold-change > 1 or < −1 and threshold as FDR < 0.05. TMM-normalized count data were obtained by utilizing Tag Count Comparison-Graphical User Interface (TCC-GUI) platform (https://infinityloop.shinyapps.io/TCC-GUI/) (Su et al., 2019). Analytical code is indicated in Figure 4—figure supplement—source data 1. We analyzed two biological replicates to show reproducibility, except E19.5 DEHP sample due to failed library preparation.

### Data analysis

The Database for Annotation, Visualization, and Integrated Discovery (DAVID v6.8, https://david.ncifcrf.gov/, Classification stringency: medium) (Huang et al., 2009) was used for functional annotation. Statistically significant (p<0.05) GO terms were filtered by BH-corrected p-values with cutoff value of FDR < 0.05. Venn diagram analysis was performed by InteractiVenn (http://www.interactivenn.net/) (Heberle et al., 2015). Heatmaps were created by using MetaboAnalyst 5.0 (http://www.metaboanalyst.ca) (Pang et al., 2021), based on DNA methylation level by RRBS and TMM-normalized count data by RNA-seq. During processing of both of RRBS and RNA-seq data, features with >50% missing values were removed, and the remaining missing values were replaced with half of the minimum positive value in the original data (default configuration). Those processed data were normalized using the auto-scaling method (mean-centered and divided by the standard deviation of each variable). Chromosome positions of hyper- or hypomethylated genes were visualized by Ensembl (https://asia.ensembl.org/index.html).

### Bisulfite sequencing analysis

Genomic DNA was isolated using phenol/chloroform extraction and ethanol precipitation. Bisulfite conversion of the DNA was performed using EZ DNA Methylation-Gold Kit (ZYMO Research D5005). Nested PCR was performed using EpiTaq HS DNA Polymerase (TaKaRa R110A). The sequences of the PCR primer were designed with MethPrimer (http://www.urogene.org/cgi-bin/methprimer/methprimer.cgi) (Li and Dahiya, 2002). The primer sequences are shown in Supplementary file 5. The PCR products were gel-purified, sub-cloned into the pGEM-T Easy vector (Promega A1360), and sequenced using an ABI PRISM 3100-Avant Genetic Analyzer (Applied Biosystems). Sequence data were analyzed with Quantification tool for Methylation Analysis (http://quma.cdb.riken.jp/top/quma_main_j.html) (Kumaki et al., 2008). The data were obtained from four and three animals in each treatment group of F1 and F2, respectively, to evaluate statistically significant differences, and data for 6–18 clones in each animal were used for analysis.

### Quantitative RT-PCR

Total RNA was extracted from cells using a RNeasy Micro Kit according to the manufacturer's instructions. RNAs were reverse-transcribed using SuperScript III reverse transcriptase (Invitrogen 18080093) and random primers (Promega C1181). Real-time PCR was performed using Power SYBR Green PCR Master Mix (Applied Biosystems 4367659). Thermal conditions were 2 min at 50°C, 10 min at 95°C, and 45 cycles of 15 s at 95°C and 60 s at 60°C. Sequences of the primers used for the PCR reaction are shown in Supplementary file 5. The *Arbp* transcript was used as an internal control. The relative expression was analyzed using comparative CT method. The data were obtained from six and three animals in each treatment group of F1 and F2, respectively, to evaluate statistically significant differences.

### Luciferase reporter assay

The gene regions between 500 bp upstream and downstream from the TSSs of *Hist1h2ba*, *Sycp1*, and *Taf7l* were amplified and sequenced. The primer sequences are shown in Supplementary file 5. Each sequence was cloned into the CpG-free pCpGL-basic Luciferase vector (Klug and Rehli, 2006). Luciferase reporter constructs were either mock-treated or methylated in vitro with SssI CpG methyltransferase for 4 hr at 37°C and purified with the QIAquick Purification Kit (QIAGEN 28704). Reporter plasmid (500 ng) and *Renilla* phRL-TK control vector (50 ng; Promega E2241) were co-transfected into HEK293T cells cultured in DMEM containing 10% FBS using Lipofectamine LTX Reagent with PLUS Reagent (Invitrogen 15338100). After 48 hr, cells were lysed, and the relative luciferase activities were analyzed using the Dual-Luciferase Reporter Assay System (Promega E1910) on a Lumat LB 9507 (Berthold). Firefly luciferase (Luc) activity of individual transfections was normalized against *Renilla* luciferase activity. We analyzed data from three independent experiments to evaluate statistically significant differences.

### Statistical analysis

The significance of difference was assessed by the unpaired two-sided Student’s t-test after confirmation of normal distribution of the data by Kolmogorov–Smirnov test, except for bisulfite sequence. The data of Kolmogorov–Smirnov test is indicated in the source data files. Statistical analysis of DNA methylation levels was performed by using the Mann–Whitney U test. The level of significance was set at p-value < 0.05.

## Data Availability

Sequencing data have been deposited in DDBJ under accession code DRA012076 and DRA012092. The following datasets were generated: MatsuiY2021Gene expression profiling of germ cells by maternal exposure to Di (2-ethylhexyl) phthalateDDBJDRA012076 MatsuiY2021DNA methylation profiling of germ cells by maternal exposure to Di (2-ethylhexyl) phthalateDDBJDRA012092

## Additional files

Supplementary file 1. Methylation levels (%) of various genomic regions.

Supplementary file 2. List of hyper- (DEHP-oil [%] > 5) and hypo- (DEHP-oil [%] < −5) methylated genes in E19.5 testicular germ cells and somatic cells for Figure 3.

Supplementary file 3. List of hyper- (DEHP-oil [%] > 5) and hypo- (DEHP-oil [%] < −5) methylated genes in spermatogonia, spermatocyte, and round spermatid for Figure 3.

Supplementary file 4. List of down- and upregulated genes in E19.5 testicular germ cells, and spermatogonia, spermatocyte, and round spermatid in adult testis (log2-fold-change > 1 or < −1, FDR < 0.05) for Figure 4—figure supplement 1.

Supplementary file 5. Primers used in this study (for Figure 2, Figure 4, Figure 6, and Figure 4—figure supplement 2).

Transparent reporting form
