# Supplementary material for: Epi-mutations for spermatogenic defects by maternal exposure to di(2-ethylhexyl) phthalate
Source: eLife. 2021 Jul 28;10:e70322. doi: 10.7554/eLife.70322 (PMC8318585; doi:10.7554/eLife.70322)
Supplement: Figure 3—source data 1. [file elife-70322-fig3-data1.docx]

**Analytical code of RRBS**

#Combine Fastq files of the same sample

$ cat O-germ1_ATCATG_L001_R1_001.fastq.gz O-germ1_ATCATG_L002_R1_001.fastq.gz > 1.fastq.gz

$ zcat 1.fastq.gz > 1.fastq

#Adaptor removal

$ cutadapt -a GATCGGAAGAGCACACG -O 10 -m 25 1.fastq -o 1c.fastq

#FastQC

$ fastqc -f fastq 1c.fastq --extract -nogroup -o 1_qc

#Removal of “CCGG” located at the head of read

$ fastx_trimmer -t 4 -i 1c.trimmed.fastq -Q 33 -o 1ct.trimmed.fastq

#Mapping

$ bismark --bowtie2 --path_to_bowtie /usr/local/bowtie2-2.0.5 /disk1/hiura/takashima/Mus_musculus/UCSC/mm10/Sequence/Bismark/ 1ct.trimmed.fastq

$ mv 1ct.trimmed.fastq_bismark_bt2.sam 2_mapping/ | mv 1ct.trimmed.fastq_bismark_bt2_SE_report.txt 2_mapping/

#Calculation of cytosine methylation

$ bismark_methylation_extractor -o ./3_report -s ./2_mapping/1ct.trimmed.fastq_bismark_bt2.sam --comprehensive --merge_non_CpG --report

$ samtools view -bS ./2_mapping/1ct.trimmed.fastq_bismark_bt2.sam > 1ct.trimmed.fastq_bismark.bam

$ samtools sort 1ct.trimmed.fastq_bismark.bam 1ct.trimmed.fastq_bismark.sort

$ samtools index 1ct.trimmed.fastq_bismark.sort.bam

$ perl /usr/local/src/bismark_v0.7.12/bismark2bedGraph CpG_context_1ct.trimmed.fastq_bismark.txt --cutoff 3 -o CpG_context_1ct_trimmed.fastq_bismark.bedgraph

#Conversion of bedgraph to bed files (RStudio in windows)

$ n1 <- read.csv("CpG_context_1ct_trimmed.fastq_bismark.bedgraph", header=F, sep="\t")

$ n2 <- read.csv("CpG_context_1ct_trimmed.fastq_bismark.bedgraph", header=F, sep="\t")

$ names(n1) <- c("V1", "V2", "V3", "methyl_germ_oil_1")

$ names(n2) <- c("V1", "V2", "V3", "methyl_germ_oil_2")

$ mix <- merge(n1, n2)

$ n1_1 <- mix[,c(1:3, 4)]

$ n2_1 <- mix[,c(1:3, 5)]

$ names(n1_1) <- c("V1", "start", "end", "methyl_germ_oil_1")

$ names(n2_1) <- c("V1", "start", "end", "methyl_germ_oil_2")

$ n1_1$start <- formatC(n1_1$start,format="d")

$ n1_1$end <- formatC(n1_1$end,format="d")

$ n2_1$start <- formatC(n2_1$start,format="d")

$ n2_1$end <- formatC(n1_1$end,format="d")

$ write.table(n1_1, "C:/Users/hiura/Desktop/RRBS_tando/tando_RRBS_bed/n1_181011.bed", sep="\t", append=F, quote=F, row.names=F, col.names=F)

$ write.table(n2_1, "C:/Users/hiura/Desktop/RRBS_tando/tando_RRBS_bed/n1_181011.bed", sep="\t", append=F, quote=F, row.names=F, col.names=F)

#Extraction of genome regions

$ dos2unix-6.0.4/dos2unix n1_181011.bed

$ bedtools-2.17.0/bin/intersectBed -a n1_181011.bed -b mm10_pro.bed -wa -wb > pro_n1_181011.bed

$ bedtools-2.17.0/bin/intersectBed -a n1_181011.bed -b mm10_body.bed -wa -wb > body_n1_181011.bed

$ bedtools-2.17.0/bin/intersectBed -a n1_181011.bed -b mm10_CGI.bed -wa -wb > CGI_n1_181011.bed

$ bedtools-2.17.0/bin/intersectBed -a n1_181011.bed -b mm10_CGIshore.bed -wa -wb > CGIshore_n1_181011.bed

$ bedtools-2.17.0/bin/intersectBed -a n1_181011.bed -b mm10_ICR.bed -wa -wb > ICR_n1_181011.bed

$ bedtools-2.17.0/bin/intersectBed -a n1_181011.bed -b mm10_SINE.bed -wa -wb > SINE_n1_181011.bed

$ bedtools-2.17.0/bin/intersectBed -a n1_181011.bed -b mm10_LINE.bed -wa -wb > LINE_n1_181011.bed

$ bedtools-2.17.0/bin/intersectBed -a n1_181011.bed -b mm10_LTR.bed -wa -wb > LTR_n1_181011.bed

$ bedtools-2.17.0/bin/intersectBed -a n1_181011.bed -b mm10_repDNA.bed -wa -wb > repDNA_n1_181011.bed

#Calculation of cytosine methylation for each promoter

$ aaa <- read.csv("pro_n1_181011.bed", header=F, sep="\t")

$ aaa$ID <- paste(aaa$V5, aaa$V6, aaa$V7, aaa$V8)

$ aaa$V1 <- "chr"

$ num <- xtabs(~ID+V1, data=aaa)

$ mean <- xtabs(V4~ID+V1, data=aaa)

$ all <- cbind(num, mean)

$ all <- as.data.frame(all)

$ names(all) <- c("V1", "V2")

$ all$methyl <- all$V2/all$V1

$ write.table(as.matrix(all), "C:/Users/Hiura/Desktop/RRBS_tando/tando_RRBS_bed/Pro_mm10/pro_n1_181011.txt", append=F, quote=F, row.names=T, col.names=F)ol.names=F)
