# Supplementary material for: Epi-mutations for spermatogenic defects by maternal exposure to di(2-ethylhexyl) phthalate
Source: eLife. 2021 Jul 28;10:e70322. doi: 10.7554/eLife.70322 (PMC8318585; doi:10.7554/eLife.70322)
Supplement: Figure 4—figure supplement 1—source data 1. [file elife-70322-fig4-figsupp1-data1.docx]

**Analytical codes of RNA-seq**

#Combine Fastq files of the same sample

$ cat E19.5_oil-1_CAGATC_L001_R1_001.fastq E19.5_oil-1_CAGATC_L002_R1_001.fastq > E19.5_O-1.fastq

#Fastqc

$ fastqc E19.5_O-1.fastq -o Fastqc

#Mapping

$ tophat2 -p 4 -G /home/common/xbio/db/iGenomes/Mus_musculus/UCSC/mm10/Annotation/Genes/genes.

gtf -o Tophat_result_ E19.5_O-1 /home/common/xbio/db/iGenomes/Mus_musculus/UCSC/mm10/

Sequence/Bowtie2Index/genome E19.5_O-1.fastq

#Count data

$ featureCounts -t exon -g gene_id -a /home/common/xbio/db/iGenomes/Mus_musculus/UCSC/mm10/Annotation/Genes/genes.gtf -o counts.txt Tophat_result_1/accepted_hits_E19.5_O-1.bam Tophat_result_2/accepted_hits_E19.5_O-2.bam Tophat_result_3/accepted_hits_E19.5_D-1.bam Tophat_result_5/accepted_hits_SPG_O-1.bam Tophat_result_6/accepted_hits_SPG_O-2.bam Tophat_result_7/accepted_hits_SPG_D-1.bam Tophat_result_8/accepted_hits_SPG_D-2.bam Tophat_result_9/accepted_hits_SPC_O-1.bam Tophat_result_10/accepted_hits_SPC_O-2.bam Tophat_result_11/accepted_hits_SPC_D-1.bam Tophat_result_12/accepted_hits_SPC_D-2.bam Tophat_result_13/accepted_hits_RS_O-1.bam Tophat_result_14/accepted_hits_RS_O-1.bam Tophat_result_15/accepted_hits_RS_D-1.bam Tophat_result_16/accepted_hits_ RS_D-2.bam

#TMM normalization (R in windows)

$ library(edgeR)

$ count<-read.table("E19.5 germ counts.txt",sep="\t",header=T,row.names=1)

$ count <- as.matrix(count)

$ group <- factor(c("Oil", "Oil", "DEHP", "DEHP"))

$ d <- DGEList(counts = count, group = group)

$ d <- calcNormFactors(d)

$ d <- estimateCommonDisp(d)

$ d <- estimateTagwiseDisp(d)

$ result <- exactTest(d)

$ table <- as.data.frame(topTags(result, n = nrow(count)))

$ write.table(table, file = "result.txt", col.names = T, row.names = T, sep = ",")
