# Supplementary material for: Epi-mutations for spermatogenic defects by maternal exposure to di(2-ethylhexyl) phthalate
Source: eLife. 2021 Jul 28;10:e70322. doi: 10.7554/eLife.70322 (PMC8318585; doi:10.7554/eLife.70322)
Supplement: Peer review history

# Decision letter

**DOI:** 10.7554/eLife.70322.sa1

**Affiliations:** University of Southern CaliforniaUnited States; Université de GeneveSwitzerland

Our editorial process produces two outputs: i) public reviews designed to be posted alongside the preprint for the benefit of readers; ii) feedback on the manuscript for the authors, including requests for revisions, shown below. We also include an acceptance summary that explains what the editors found interesting or important about the work.

**Acceptance summary:**

Maternal exposure to a certain phthalate (DEHP) has been shown to cause spermatogenesis defects in the male progeny, and in their offspring. In this manuscript, Tando et al. have investigated the molecular consequences of this maternal exposure on fetal and adult male germ cells by studying DNA methylation and gene expression by large-scale approaches. They found three genes previously known to be involved in spermatogenesis that are deregulated following maternal exposure to DEHP and which could contribute to the observed spermatogenesis defects.

**Decision letter after peer review:**

Thank you for submitting your article "Epi-mutations for spermatogenic defects by maternal exposure to Di (2-ethylhexyl) phthalate" for consideration by *eLife*. Your article has been reviewed by 3 peer reviewers, one of whom is a member of our Board of Reviewing Editors, and the evaluation has been overseen by Jessica Tyler as the Senior Editor. The following individual involved in review of your submission has agreed to reveal their identity: Ludwig Stenz (Reviewer #2).

The reviewers have discussed their reviews with one another, and the Reviewing Editor has drafted this to help you prepare a revised submission.

Essential Revisions:

1. The reviewers had concerns about the robustness and clarity of the statistical analyses that need to be addressed in a revised manuscript. This includes (i) much more detailed description and clarifications on the statistical methods (i.e. inclusion of software version numbers, FDR cutoffs, etc.), (ii) need to revise any analysis relying on the deprecated use of the FPKM framework, (iii) use of Student t-test in the absence of proof of normal distribution.

2. There were some concerns about the small number of replicates used (n = 2), reduced to n=1 for one type (E19.5 DEHP RNAseq analysis). This small number of replicates provide extremely limited statistical power and thus will make it difficult to capture the extent of biological variation (potentially missing many true targets). The ideal solution, which the reviewers recognize may be too time-consuming and difficult to achieve right now, would be to add 2 additional replicates to these analyses (so as to be able to account for batch effects). However, if this is too difficult to achieve, the reviewers recommend that all general conclusions be strongly toned down in the abstract, text and discussion to reflect that there is strong statistical support only for 3 targets that are further validated (Hist1h2ba, Sycp1, and Taf7l). The limitation due to the small sample number should also be explicitly discussed.

3. Please clarify other reviewer concerns that may affect understanding/interpretation/reproducibility of results: Reviewer #2 point on Hoechst blue/Hoechst red, reviewer #3 point on the unusual GFRA1 signal in spermatocytes.

*Reviewer #1 (Recommendations for the authors):*

1. RNA-seq data need to be reanalyzed as described in the Public Review.

2. The Materials and methods section on RRBS analysis and RNA sequencing should include a detailed description on how significant regions or genes were defined (i.e. FDR significance threshold, etc.). The thresholds for false discovery rate should also be clearly indicated for the DAVID analysis (currently only p-values are indicated in Figure 2).

3. For long-term reproducibility of analyses, all version numbers of all software and packages should be provided in the Material and Methods (e.g. Tophat2, Bowtie, Cuffdiff, etc.). In addition, analytical code should be made available either as a Supplementary file or through a Github repository. Finally, all accession information should be available – it was not at the time of review (see lines 501-504).

4. Authors used unpaired two-sided Student's t-test to calculate statistical significance for a number of assays, including those in Figure 1C and Figure Supplement 1C. As a t-test can be applied when the dataset follows a normal distribution, a normality test should be performed and indicated. If normal distribution of the data points cannot be shown, a nonparametric statistical test (e.g.Wilcoxon test).

*Reviewer #2 (Recommendations for the authors):*

1) The figure supplement 2 is of high importance for understanding the cells purification process and should be more up in the manuscript instead of being a supplement; it is one of the key technical achievement of the work.

a) GFP-positive germ cells were extracted from adult by using a transgenic mice with GFP green produced under the control of the Oct4 promoter, Oct4 being a germ cell marker. That aspect is very clear. However, a breeding schema will be a plus for the reader to get a clear vision of the work. Panel A show how few the germ cells are in F1 embryos according to the Oct4-FACS sorting from the transgenic mice. This probably explain why the authors had to pool different experiments producing only two replicates.

b) The germ cells sub-population extracted from adult testis used another less clear approach (using propidium iodide, a red marker of death and Hoechst staining DNA in blue). Panel B of the Figure strangely mention Hoechst blue in one axe and Hoechst red in the other and propidium iodide appears in the material section, as if it was a mistake somewhere. Moreover, I do not understand really if the transgenic mice were also used for F1 adult and how GFP may interfere with the red and blue emission used to extract the cells.

c) The legend of the figure mention "C.D." but there is no panel D.

2) In the first paragraph of the results, tubules divided by the presence of vacuoles, which appear associated with defect in germ cells.

a) I think that germ cells default is the important aspect of the work, instead of vacuole. (i.e. "in tubule with large vacuole, germ cells were typically deleted" → tubule without germ cells contains large vacuole…). That explain the decreased sperm counts we and other observed in F1 male after prenatal exposure to DEHP. Please illustrate the vacuole in the different type of tubule with arrow in Figure 1.

b) Apoptotic cells detection required Tunnel assay.

3) For the heat map, such as in Figure 2 panel A, please explain the sense of the colors blue and red, as reflecting the sense of the promoter methylation changes.

4) Figure supplement 3, mention testicular somatic cells instead of somatic cells.

*Reviewer #3 (Recommendations for the authors):*

My main concern is the small number of replicates. At least a third replicate per sample type would be needed to produce an accurate list of DMRs and DEGs. Detailed statistical analyses should also be presented.

Another concern is that no statistical difference was found for the F2. I therefore don't think it is accurate to claim in the article that "Expression and methylation of those genes tended to be downregulated and increased, respectively in F2 spermatogonia following maternal DEHP exposure." (abstract and discussion).

Finally, can the authors explain why GFRA1 signal is strong in spermatocytes – as it is normally a spermatogonia marker?

# Reply: Author response

**DOI:** 10.7554/eLife.70322.sa2

Essential Revisions:

1. The reviewers had concerns about the robustness and clarity of the statistical analyses that need to be addressed in a revised manuscript. This includes (i) much more detailed description and clarifications on the statistical methods (i.e. inclusion of software version numbers, FDR cutoffs, etc.), (ii) need to revise any analysis relying on the deprecated use of the FPKM framework, (iii) use of Student t-test in the absence of proof of normal distribution.

According to the suggestion, we added detailed statistical methods including software version numbers, FDR cutoffs in Materials and methods and figure legends (page 19, 20, 30, 32). We re-estimated gene expression levels form the RNA-seq data by TMM normalization (page 20). We confirmed normal distribution of data points for unpaired two-sided Student’s t-test, and the results were shown in Figure 3-source data 1, Figure 4—figure supplement 1-source data 1 files, and the source data files for each figure, and were mentioned it in Materials and methods (page 19, 20, 22).

2. There were some concerns about the small number of replicates used (n = 2), reduced to n=1 for one type (E19.5 DEHP RNAseq analysis). This small number of replicates provide extremely limited statistical power and thus will make it difficult to capture the extent of biological variation (potentially missing many true targets). The ideal solution, which the reviewers recognize may be too time-consuming and difficult to achieve right now, would be to add 2 additional replicates to these analyses (so as to be able to account for batch effects). However, if this is too difficult to achieve, the reviewers recommend that all general conclusions be strongly toned down in the abstract, text and discussion to reflect that there is strong statistical support only for 3 targets that are further validated (Hist1h2ba, Sycp1, and Taf7l). The limitation due to the small sample number should also be explicitly discussed.

We agree that two biological replicates for RNA-seq and RRBS are not enough to show a global effect of maternal DEHP exposure on gene expression and DNA methylation. Because it is time-consuming and difficult to achieve additional experiments to obtain more of replicates, we toned down general conclusions and discussions concerning genome-wide consequences of the DHEP exposure as suggested, and focused on the three target genes that were extracted from the genome-wide data (Abstract, page 6, 7, 8, 10, 12). Limitation of the genome-wide analysis due to small sample number in this study was also discussed (page 7).

3. Please clarify other reviewer concerns that may affect understanding/interpretation/reproducibility of results: Reviewer #2 point on Hoechst blue/Hoechst red, reviewer #3 point on the unusual GFRA1 signal in spermatocytes.

We explained Hoechst/propidium iodide staining for testicular cell fractionation in more detail in Materials and Method (page 17), and also explained GFRA1 signal in spermatocyte in Result (page 6).

Reviewer #1 (Recommendations for the authors):

1. RNA-seq data need to be reanalyzed as described in the Public Review.

As described above, we re-estimated gene expression levels form the RNA-seq data by TMM normalization.

2. The Materials and methods section on RRBS analysis and RNA sequencing should include a detailed description on how significant regions or genes were defined (i.e. FDR significance threshold, etc.). The thresholds for false discovery rate should also be clearly indicated for the DAVID analysis (currently only p-values are indicated in Figure 2).

Because we did not apply FDR cutoff in the original manuscript, we re-analyzed RNA-seq data, and showed revised results with FDR<0.05 in the new Figure 4—figure supplement 1, and described it in Materials and methods, page 20. In this case, functional annotation by DAVID did not show spermatogenesis- and spermiogenesis-related GO terms, while sperm motility-related GO terms were enriched in the up-regulated genes in spermatogonia and spermatocytes. We edited corresponding discussion concerning possible influence of DEHP on abnormal up-regulation of genes (page 7). In the case of RRBS, we extracted differentially methylated regions (DMRs) based on 5% changes of methylation levels in DEHP-treated samples compared to oil-treated samples, which is not fully reliable, but FDR<0.05 did not result in extraction of spermatogenesis-related genes including Hist1h2ba, Sycp1, and Taf7l. We consider the extraction of DMRs as a step for selecting candidate spermatogenesis-related genes with DMRs. We therefore left the RRBS results in new Figure 3 as it was in the original manuscript except functional annotation of differentially methylated genes, in which FDR<0.05 cutoff was applied (page 20), and toned down conclusions and discussions concerning genome-wide consequences of the DHEP exposure (Abstract, page 6, 7, 8, 10, 12), and focused on the three target genes that were extracted from the genome-wide data.

3. For long-term reproducibility of analyses, all version numbers of all software and packages should be provided in the Material and Methods (e.g. Tophat2, Bowtie, Cuffdiff, etc.). In addition, analytical code should be made available either as a Supplementary file or through a Github repository. Finally, all accession information should be available – it was not at the time of review (see lines 501-504).

We added information for analysis of RNA-seq and RRBS data, including version number of the software in the Material and Methods (page 19, 20). We also showed analytical codes in Figure 3-Source data 1 and Figure 4—figure supplement 1-Source data 1 files, and accession number of the sequence data (page 22).

4. Authors used unpaired two-sided Student's t-test to calculate statistical significance for a number of assays, including those in Figure 1C and Figure Supplement 1C. As a t-test can be applied when the dataset follows a normal distribution, a normality test should be performed and indicated. If normal distribution of the data points cannot be shown, a nonparametric statistical test (e.g.Wilcoxon test).

We confirmed normal distribution of data points for unpaired two-sided Student's t-test, which was mentioned in Materials and methods (page 22) and indicated in the Source data file for each figure.

Reviewer #2 (Recommendations for the authors):

1) The figure supplement 2 is of high importance for understanding the cells purification process and should be more up in the manuscript instead of being a supplement; it is one of the key technical achievement of the work.

a) GFP-positive germ cells were extracted from adult by using a transgenic mice with GFP green produced under the control of the Oct4 promoter, Oct4 being a germ cell marker. That aspect is very clear. However, a breeding schema will be a plus for the reader to get a clear vision of the work. Panel A show how few the germ cells are in F1 embryos according to the Oct4-FACS sorting from the transgenic mice. This probably explain why the authors had to pool different experiments producing only two replicates.

b) The germ cells sub-population extracted from adult testis used another less clear approach (using propidium iodide, a red marker of death and Hoechst staining DNA in blue). Panel B of the Figure strangely mention Hoechst blue in one axe and Hoechst red in the other and propidium iodide appears in the material section, as if it was a mistake somewhere. Moreover, I do not understand really if the transgenic mice were also used for F1 adult and how GFP may interfere with the red and blue emission used to extract the cells.

c) The legend of the figure mention "C.D." but there is no panel D.

According to the suggestion, the figure supplement 2 was moved to a new Figure 2, and a breeding schema was added. In addition, the number of germ cells and F1 embryos were indicated in the Materials and methods (page 16). Fluorescence of Hoechst 33342 has two emission wave length (450nm and 675 nm), and the stained cells can be separated by signal intensity for the two wavelengths, which partly reflects DNA content of the cells, and the FACS plot in new Figure 2C shows a staining pattern of testicular cells by the Hoechst dye. Propidium iodide used for eliminating dead cells, also has 675 nm emission, but the stained cells were eliminated by pre-gating. Influence of GFP fluorescence on Hoechst fluorescence was eliminated by the compensation setting of the flow cytometer. We added related descriptions in Materials and methods (page 17).

2) In the first paragraph of the results, tubules divided by the presence of vacuoles, which appear associated with defect in germ cells.

a) I think that germ cells default is the important aspect of the work, instead of vacuole. (i.e. "in tubule with large vacuole, germ cells were typically deleted" → tubule without germ cells contains large vacuole…). That explain the decreased sperm counts we and other observed in F1 male after prenatal exposure to DEHP. Please illustrate the vacuole in the different type of tubule with arrow in Figure 1.

b) Apoptotic cells detection required Tunnel assay.

We edited description concerning vacuoles (page 4), and pointed put the vacuoles by arrows in Figure 1A, B, and Figure 1—figure supplement 1A, B. To confirm apoptotic cells, we stained the testis sections by the antibody against active Caspase 3, and added the result in Figure 1A.

3) For the heat map, such as in Figure 2 panel A, please explain the sense of the colors blue and red, as reflecting the sense of the promoter methylation changes.

We added sentences describing relationship between the sense of colors and the sense of the expression and methylation changes in the legends of new Figure 3 and new Figure 4—figure supplement 1.

4) Figure supplement 3, mention testicular somatic cells instead of somatic cells.

We edited the legend of new Figure 3—figure supplement 1 as suggested.

Reviewer #3 (Recommendations for the authors):

My main concern is the small number of replicates. At least a third replicate per sample type would be needed to produce an accurate list of DMRs and DEGs. Detailed statistical analyses should also be presented.

We agree that two biological replicates for RNA-seq and RRBS are not enough to show a global effect of maternal DEHP exposure on gene expression and DNA methylation. Because it is time-consuming and difficult to achieve additional experiments to obtain more of replicates, we toned down general conclusions and discussions concerning genome-wide consequences of the DHEP exposure as suggested (Abstract, page 6, 7, 8, 10, 12), and focused on the three target genes that were extracted from the genome-wide data. Limitation of the genome-wide analysis due to small sample number in this study was also discussed (page 7). We also described statistical methods in Materials and methods (page 20).

Another concern is that no statistical difference was found for the F2. I therefore don't think it is accurate to claim in the article that "Expression and methylation of those genes tended to be downregulated and increased, respectively in F2 spermatogonia following maternal DEHP exposure." (abstract and discussion).

As pointed out, changes of DNA methylation and expression of the three candidate genes by DEHP in F2 are not statistically significant, and we further toned down the effects of DEHP in F2 spermatogonia (Abstract, page 9, 12).

Finally, can the authors explain why GFRA1 signal is strong in spermatocytes – as it is normally a spermatogonia marker?

As pointed out, GFRA1 is a spermatogonia marker, and its signal in spermatocytes may be due to contamination of spermatogonia in the spermatocyte fraction. We mentioned it in Result (page 6).
